# High‐resolution gene expression atlases of two contrasting major Greek olive (*Olea europaea* L.) tree cultivars for oil and table olive production

**DOI:** 10.1111/ppl.14600

**Published:** 2024-11-05

**Authors:** Georgios Lagiotis, Ioanna Karamichali, Maria Astrinaki, Androniki C. Bibi, Despoina Vassou, Georgia‐Maria Nteve, Anastasios Kollias, Ioanna Manolikaki, Christina Skodra, Michail Michailidis, Maria Manioudaki, Marios Iakovidis, Ioannis Ganopoulos, Georgios Koubouris, Athanassios Molassiotis, Christos Bazakos, Dimitris Kafetzopoulos, Panagiotis Madesis

**Affiliations:** ^1^ Centre for Research and Technology Hellas (CERTH) Institute of Applied Biosciences (INAB) Thessaloniki Greece; ^2^ Institute of Molecular Biology and Biotechnology (IMBB) Foundation for Research and Technology – Hellas (FORTH) Heraklion Greece; ^3^ Institute of Olive Tree Subtropical Crops and Viticulture Chania Greece; ^4^ Laboratory of Pomology, Department of Horticulture Aristotle University of Thessaloniki Thessaloniki‐Thermi Greece; ^5^ Department of Informatics and Computer Engineering University of West Attica Egaleo Greece; ^6^ Institute of Plant Breeding and Genetic Resources Hellenic Agricultural Organization ELGO‐DIMITRA Thessaloniki Greece; ^7^ Joint Laboratory of Horticulture ELGO‐DIMITRA Thessaloniki‐Thermi Greece; ^8^ Department of Comparative Development and Genetics Max Planck Institute for Plant Breeding Research Cologne Germany; ^9^ Laboratory of Molecular Biology of Plants, School of Agricultural Sciences University of Thessaly Volos Greece

## Abstract

**Description of aims and systems used:**

Olive (*Olea europea* L.) is one of the most economically important tree crops worldwide, especially for the countries in the Mediterranean basin. Given the economic and nutritional importance of the crop for olive oil and drupe production, we generated transcriptional atlases for the Greek olive cultivars “Chondrolia Chalkidikis” and “Koroneiki” which have contrasting characteristics in terms of fruit development, oil production properties, and use. Our analysis involved 14 different organs, tissue types, and developmental stages, including young and mature leaves, young and mature shoots, open and closed flowers, young and mature fruits (epicarp plus mesocarp), young and mature endocarps, stalks, as well as roots. The developed gene expression atlases and the associated resources offer a comprehensive insight into comparative gene expression patterns across several organs and tissue types between significant olive tree cultivars. The comparative analyses presented in this work between the “Koroneiki” cultivar, which performs better in olive oil production, and the “Chondrolia Chalkidikis,” which grows larger fruits, will be essential for understanding the molecular mechanisms underlying olive oil production and fruit shape and size development. The developed resource is also expected to support functional genomics and molecular breeding efforts to enhance crop quality and productivity in olive trees.

**Outline of data resources generated:**

The transcriptome data were generated using paired‐end Illumina Next‐Generation Sequencing technologies. The sequencing yielded approximately 13 million reads per sample for “Chondrolia Chalkidikis” and around 24 million reads per sample for “Koroneiki.” The transcriptomes were comparatively analyzed to reveal tissue‐specific and differentially expressed genes and co‐expression gene modules within and between cultivars.

**Summary of key results:**

The comparative analysis unveiled tissue‐specific and differentially expressed genes within and between cultivars. Hierarchical gene clustering revealed intra‐ and inter‐cultivar expression patterns, particularly for the endocarp and fruit tissues relevant to olive oil production and fruit development. Additionally, genes associated with oil production and fruit size/shape development, including those in fatty acid metabolism and developmental regulation, were identified.

**Broader utility of the resource:**

To facilitate accessibility, the GrOlivedb (www.GrOlivedb.com) database was developed, housing the comprehensive transcriptomic data for all of the analyzed organs and tissue types per cultivar. This resource will be a useful molecular tool for future breeding studies in olive oil production and fruit development and a valuable resource for crop improvement.

## 1 INTRODUCTION

The olive tree (*Olea europaea* L.) is one of the oldest tree crops in the world and the most iconic plant of the Mediterranean basin (Kapellakis et al., 2008; Besnard & Rubio de Casas, 2016). The species belongs to the Oleaceae family, which comprises 28 extant genera with a total estimate of 700 species (*Olea europaea L. | Plants of the World Online | Kew Science*, 2022). According to contemporary classifications, two taxonomic varieties of *O. europaea* L. are recognized: the domesticated variety *O. europaea* var. europaea and the wild variety *O. europaea* var. sylvestris, which is considered the ancestral form to the olive cultivars (Khadari et al., 2008). It has been recently demonstrated that olive tree domestication involved a major domestication event and subsequent recurring admixtures of the domesticated cultivars with wild populations across the Mediterranean Basin (Julca et al., 2020). Although the olive tree is primarily grown in the Mediterranean basin, its cultivation has been spread to other parts of the world that are characterized by mild climate conditions (Besnard et al., 2009).

Over the last decade, olive cultivation in Europe covered an average of 4 920 608 hectares of land, corresponding to ~48% of the worldwide cultivation (FAOSTAT, 2022). Within Europe, countries like Greece, Italy, and Spain have traditionally been the leading producers of olive and olive‐related products (Vossen, 2007). Amongst the various olive‐derived products, the tree is primarily cultivated for its oil and table olives, as well as for recreational and environmental purposes (Bartolini et al., 1998). Olive oil, one of the most economically important olive products, is rich in secondary metabolites with health‐promoting properties, such as tyrosol, hydroxytyrosol, and oleuropein (Tuck & Hayball, 2002). Its consumption has been associated with preventing and treating several cardiovascular diseases and certain types of cancer (Pauwels, 2011). Apart from olive oil, table olives are also a product of high economic value, and their consumption was also shown to have health‐promoting properties (Raederstorff, 2009).

To improve molecular breeding, with the aim of increasing crop quality and productivity, the enrichment of genetic resources with effective molecular tools is essential, especially for non‐model crop plants like olive trees. The implementation of high‐throughput sequencing technologies enabled the generation of genome assemblies for wild and domesticated olive tree cultivars, which have provided valuable insights into the domestication and evolution of olive trees and the genetic structure of their genomes (Unver et al., 2017; Jiménez‐Ruiz et al., 2020; Julca et al., 2020; Rao et al., 2021). Next‐generation sequencing technologies have also been implemented to generate olive transcriptomes from various organs and stress responses and discriminate varieties (Muleo et al., 2016; Skodra et al., 2021). Most of the generated olive transcriptomes were used to investigate flower and fruit development, given the importance of the organs in drupe and olive oil production (Alagna et al., 2009, 2016; Muñoz‐Mérida et al., 2013; Carmona et al., 2015; Iaria et al., 2016; Salimonti et al., 2021). Apart from fruit and flower development, transcriptomic data were also generated for studying the process of fruit abscission in the Spanish “Picual” cultivar (Gil‐Amado & Gomez‐Jimenez, 2012; Parra et al., 2013), fruit loading in the “Conservolia” cultivar (Dastkar et al., 2020), the altitudinal effects on olive quality (Bruno et al., 2019), as well as for understanding abiotic stress responses in the cultivars “Kalamon” (Bazakos et al., 2015), “Leccino” (Guerra et al., 2015), and “Picual” (De La O Leyva‐Pérez et al., 2015). A combination of transcriptomics and other omics analyses was also used to holistically understand drupe biology and ripening mechanisms (Biton et al., 2015; Xiaoxia et al., 2020). Recently, our consortium reported the first proteogenomic analysis for understanding the changes occurring in roots and leaves of the cultivar “Chondrolia Chalkidikis”, challenged with salinity priming (Skodra et al., 2022). Despite the latest advancements in olive research and the implementation of high‐throughput next‐generation sequencing technologies in olive biology, a comprehensive atlas of global gene expression for olive is still missing. Notably, most transcriptomic studies in olive tree have focused on either selected tissue types that fail to provide the necessary resolution, primarily, fruit and flower organs, or whole‐organ analyses. Furthermore, investigating the molecular basis of olive oil production and fruit shape/size development necessitates a more thorough approach, utilizing comparative transcriptomic data generated from various organs and tissue types between olive cultivars with contrasting characteristics.

Aiming to address these issues and generate a tool for facilitating comparative research in olive oil production and development, gene expression atlases were developed in this study for two iconic Greek olive cultivars of high economic value, namely “Chondrolia Chalkidikis” (CHO) that is mainly grown for table olives, and “Koroneiki” (KO) for olive oil production. The analysis involved 14 organs, tissue types, and developmental stages per cultivar, including various leaves, shoots, flowers, fruits, and endocarp developmental stages, as well as stalks and roots. Illumina RNA‐seq data were analyzed to unravel gene expression profiles across different organs, tissues, and developmental stages within and between cultivars. Moreover, the GrOlivedb database (http://www.grolivedb.com/) was also developed for collectively accessing both raw and processed transcriptomic data for each individual sample of both olive tree cultivars. The present gene expression atlases will not only provide a comprehensive view of global gene expression in major olive tissue types and developmental stages, especially for olive cultivars with contrasting characteristics in terms of oil production and fruit development but will also facilitate the application of functional genomics and molecular breeding in olive trees.

## 2 MATERIALS AND METHODS

The following section summarises the materials and methods used in this work. Detailed descriptions and additional results are in the supplementary materials (Supplementary File 1). Plant tissues were collected from Greek olive tree cultivars that had been morphologically and genetically characterized as *Olea europaea* L. cv. “Chondrolia Chalkidikis” (CHO) and *O. europea* L. cv. “Koroneiki” (KO). Our analysis involved 14 organs, tissue types, and developmental stages per cultivar, including i) young and mature leaves, ii) young and mature shoots, iii) closed and open flowers, iv) various fruits (epicarp and mesocarp) developmental stages (stages 2–4), v) several endocarp developmental stages (stages 2–4), vi) stalks, and v) roots, hereunder referred to as tissue types for simplicity (Supplementary Table 1). Tissue collection, RNA extraction, and RNA sequencing library preparations were performed in three biological replicates. The transcriptome data was generated using paired‐end Illumina Next‐Generation Sequencing technologies. The raw sequencing reads were filtered, removing low‐quality reads and adaptor sequences, and were subsequently mapped to the wild olive (*O. europaea* var. *sylvestris*) reference genome (accession no. MSRW00000000; BioProject record ID PRJNA350614; (Unver et al., 2017).

The transcriptome data per tissue type and cultivar were further analyzed to generate the gene specificity and differentially expressed genes (DEGs) datasets. Regarding tissue specificity, the mean values of the filtered gene raw counts were used to evaluate the expression specificity of the genes in the entirety of all grouped samples per tissue and cultivar, but also between the tissues of a specific cultivar or between cultivars using the *tau* index of gene specificity (Yanai et al., 2005). The specificity of each gene per individual tissue or cultivar was calculated using the *tau* expression fraction (*tef*). For generating the DEGs datasets, the raw read counts per tissue type, developmental stage and cultivar were normalized to facilitate sample comparisons, utilizing the “median ratio method” (Anders & Huber, 2010). DEG comparisons were performed for the different tissues within and between cultivars. Volcano plots of the DEG datasets were generated by plotting the Log_2_ Fold Change (FC) on the x‐axis against the p‐value on the y‐axis (*p*_*adj*_ <0.05 was used as the statistical significance cutoff value).

A hierarchical gene clustering approach (Reynolds et al., 2006) was performed to unravel intra‐ and inter‐cultivar gene expression patterns in the endocarp and fruit tissues, which are directly associated with olive oil and drupe production. Changes in gene expression patterns were investigated at different developmental stages in each tissue type per cultivar and at the same developmental stage for each tissue between cultivars. The parameters used were the Euclidean distance metric to calculate a “distance” metric between each pair of genes and the complete linkage clustering method to cluster the genes hierarchically. The number of clusters was set to either four or six (*p*_*adj*_ <0.01) based on the formation of discernible patterns between cultivars and enrichment in genes involved in oil production and fruit development (Supplementary Data S8).

To identify genes involved in oil production and fruit development, the highly specific gene datasets (Supplementary Data S3), the CHO vs. KO DEG datasets for the flower, endocarp, and fruit tissues (Supplementary Data S4), as well as the most significantly enriched CHO/KO co‐expression gene clusters (Supplementary Data S6 and S8) were surveyed for the presence of “known” genes with documented function in these processes (Supplementary Data S9 and S10). Several of these datasets were also analyzed with functional GO (Gene Ontology) enrichment analysis, using the *Olea europaea* var. *sylvestris* gene annotations, including KEGG pathway options, to reveal the most significantly enriched biological processes. Comparative qRT‐PCR was also performed to verify the RNA seq results by analyzing five of the most significant and highly expressed CHO vs. KO DEGs with reported involvement in oil production (Supplementary File 1). The reactions for each gene per cultivar and tissue type were performed in triplicates. The *Olea europaea* L. polyubiquitin (*OUB2*) was used as an internal reference gene (Ray & Johnson, 2014). Sample calibration was performed using either the CHO Endocarp stage 2 or the CHO Fruit Stage 3 for normalization.

Finally, a dedicated database (GrOlivedb; https://grolivedb.com) was developed, presenting the transcriptomic data for each individual tissue of both Greek olive tree cultivars. The GrOlivedb is a web‐based, curated, relational database that builds upon the open‐source Drupal content management system and the GMOD Chado database schema (Mungall et al., 2007). The developed database provides access to the raw data submitted in GenBank (BioProject ID: PRJNA763324; https://www.ncbi.nlm.nih.gov/bioproject/?term=Olea) and the most significant data generated in this work (Supplementary Data [Link], [Link], and S6). NCBI BLAST sequence similarity search and tissue expression visualization tools have also been implemented.

## 3 RESOURCE OVERVIEW

The olive tree is one of the most economically important oil‐producing tree crops in the world, especially for the countries in the Mediterranean basin (Kapellakis et al., 2008). Given their importance for oil and table olives production (Delgado et al., 2016), we generated the first transcriptional atlases for two iconic Greek olive tree cultivars, namely “Chondrolia Chalkidikis” (CHO) and “Koroneiki” (KO), with contrasting characteristics in terms of fruit development and oil production. The comprehensive transcriptomic analysis involved 14 tissue types and developmental stages, including fruits, endocarp, flowers, leaves, shoots, stalks, and root tissues for both cultivars (Supplementary Table 1).

The generated transcriptomes were comparatively analyzed to reveal tissue‐specific and differentially expressed genes for each tissue type within and between cultivars (Supplementary Data [Link], [Link]). A hierarchical gene clustering approach was also performed to unravel any intra‐ and inter‐cultivar gene expression patterns in the endocarp and fruit tissues directly associated with olive oil and drupe production (Supplementary Data [Link], [Link]). To this end, the generated olive transcriptome datasets were further surveyed for genes previously reported to play a role in oil production, fruit size, and shape development (Supplementary Data [Link], [Link]). The intersection of these gene sets with the fruit, endocarp, and flower DEGs datasets revealed a plethora of constitutive and novel genes with immense research potential (Supplementary Data S11).

Moreover, the GrOlivedb database (www.GrOlivedb.com) was developed for collectively accessing both raw and processed transcriptomic data for each individual tissue type of both olive tree cultivars. The GrOlivedb database implements gene code (NCBI GenInfo Identifier) and nucleotide sequence‐based gene search tools (Expression and Blastn) with visualization capabilities. Overall, the developed gene expression atlases and the associated resources offer an extensive insight into comparative gene expression patterns across significant olive tissue types and cultivars. The value of the developed resource goes beyond a mere overview of gene expression, as it is poised to support functional genomics, especially for olive oil production and fruit development, and the advancement of molecular breeding efforts aimed to enhance crop quality and productivity in olive trees.

## 4 RESULTS AND DISCUSSION

### 4.1 Transcriptome quality assessment

The raw transcriptomic data of the 42 cDNA libraries corresponding to 14 tissue types and developmental stages for each of the two Greek olive cultivars (Supplementary Table 1) were filtered to an average of 13.5 million reads per sample for CHO and 24.4 million reads per sample for KO, after removing adapter sequences and low‐quality reads (average Q30 percentage 94%; Supplementary Data S1). The filtered transcriptome was mapped on the wild olive reference genome with an average mapping rate of 82% for both CHO and KO, with ranges between 63–93% and 80–85% per sample, respectively. Detailed mapping stats can be found in Supplementary Data S2. Although new versions of the reference genome for the domesticated olive *O. europaea var. europaea* have been released (Jiménez‐Ruiz et al., 2020; Julca et al., 2020; Rao et al., 2021), the wild variety genome was used, given test alignments of indicative CHO and KO tissues on the FARGA Oe9 domesticated olive genome showed high variation amongst the CHO and KO cultivars (Supplementary Table 2). Furthermore, the higher percentage of duplications in the FARGA Oe9 genome could significantly affect the gene counts observed.

### 4.2 Global gene expression profiles of two local olive tree cultivars

**FIGURE 1:**
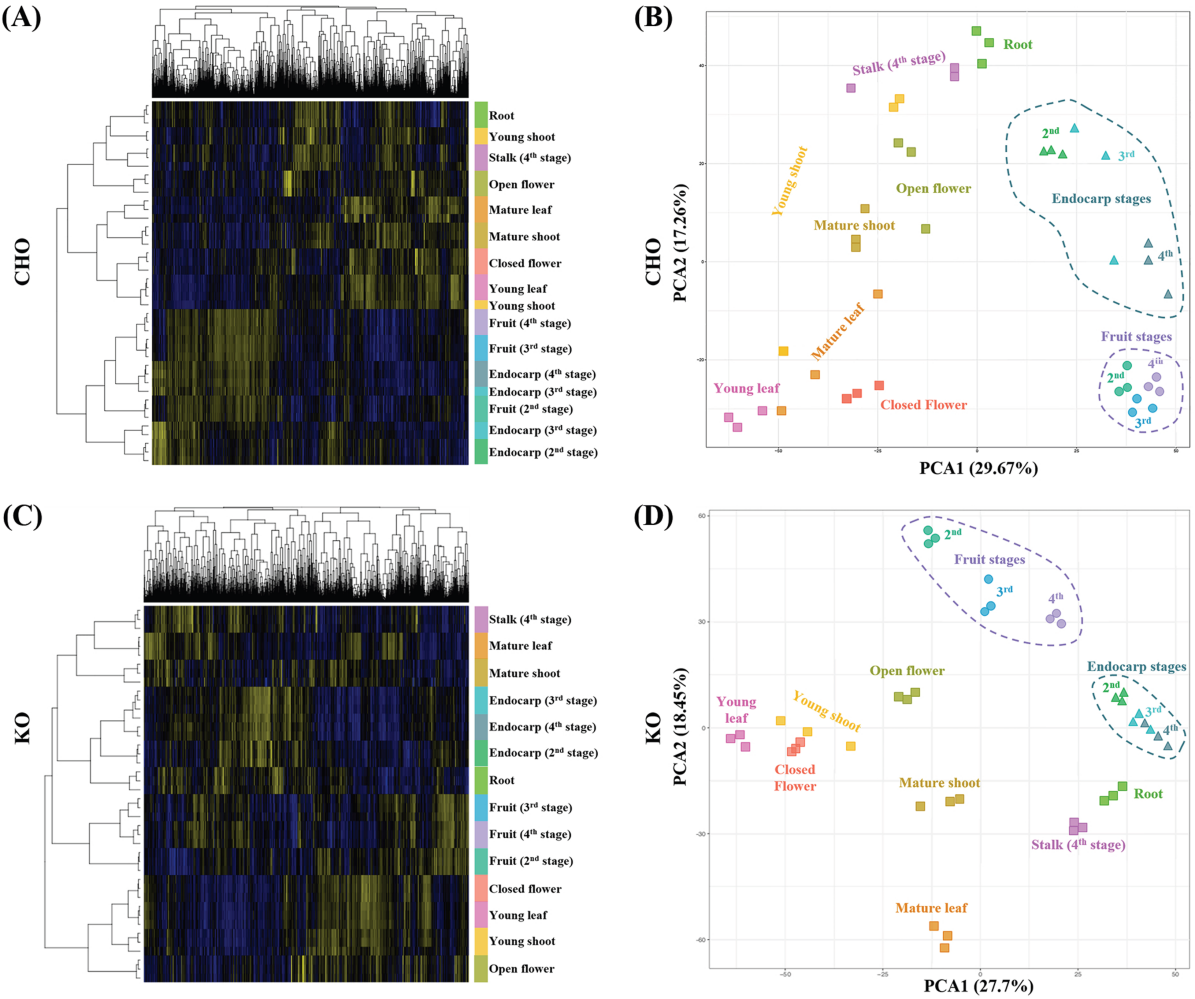
Global gene expression patterns of two Greek olive tree cultivars. (A, C) Hierarchical clustering heatmaps and (B, D) Principal Component Analysis (PCA) of gene expression amongst 14 tissue types/ developmental stages for the CHO (A and B) and KO (C and D) cultivars. The % variation explained by each axis of the PCA is indicated in parentheses. The triplicates are shown independently for each tissue type/ developmental stage per cultivar.

To investigate the global transcriptome differences amongst the various tissue types and developmental stages of CHO and KO cultivars, the Log_2_‐ transformed TPM (Transcript per Million mapped reads) data were hierarchically clustered across all samples, and Principal Component Analysis (PCA) was performed (Figure 1). The hierarchical clustering of the CHO gene expression separates the samples into two major clades, one containing the fruit and endocarp samples and another that includes the rest of the tissue types (Figure 1A). Within the fruit‐endocarp clade, both tissue types are clearly distinct, although some overlap in gene expression between the fruit samples and the final endocarp stage (stage 4) has been observed (Figure 1A). Conversely, in the other main clade that contains the root and foliar tissue samples, the various ontogenetic stages of most of the tissue types are clearly separated, indicating dramatic shifts in gene expression (Figure 1A). Similar clustering patterns have also been observed in the PCA, with the characteristic separation of the fruit and endocarp samples from the rest of the tissues primarily along the first principal component (PC1, explaining 29.67% of the observed variation; Figure 1B).

Gene expression amongst the KO tissues was less consolidated, as shown by the formation of four major clades in the hierarchical clustering analysis (Figure 1C), comprising: i) the mature leaf and shoot, as well as the stalk samples, ii) the endocarp samples and the root, iii) the fruit stages, and iv) the young leaves and shoots, as well as the flower samples (Figure 1C). Similar clustering patterns were also observed in the PCA of the KO samples (Figure 1D). More specifically, fruit and endocarp samples of the KO cultivar are characteristically separated from the foliar and root tissues (Figure 1D), similar to what was observed in the PCA of the CHO cultivar (Figure 1B). However, the KO endocarp stages have a more uniform gene expression showing tight clustering compared to the fruit stages, which show a looser clustering along the first principal component (PC1, explaining 27.7% of the observed variation; Figure 1D). In contrast, the corresponding tissue types in CHO show the reverse clustering pattern (Figure 1B).

PCA was also performed for all samples from both cultivars, which revealed interesting clustering patterns (Supplementary Figure 1). Within each cultivar, the variation observed amongst the CHO replicates (Figure 1B) was less pronounced, even amongst the young shoot and stalk samples. In contrast, KO samples' replicates remained tightly clustered (Supplementary Figure 1). Concerning inter‐cultivar variations, the samples of both cultivars showed tight clustering within each tissue‐type category, apart from young shoot, young leaf, endocarp stage 2 and Stage 3, and fruit stage 2 and Stage 3 tissues (Supplementary Figure 1). This trend was remarkably pronounced for the fruit stages 2 and 3, which exhibited loose clustering along the first principal component (PC1, explaining 47% variance; Supplementary Figure 1), indicating that CHO and KO early fruit stages are distinct. Notably, this distinction was even more pronounced when comparing mature endocarp and fruit samples (stage 4) from the less mature stages (stages 2 and 3) in both cultivars (Supplementary Figure 1), suggesting a significant shift in gene expression in these mature tissues. Nevertheless, this pattern is only observed when both CHO and KO samples are analyzed (Supplementary Figure 1) and not in the individual PCAs, in which endocarp and fruit samples are more tightly grouped (Figure 1). This is likely the result of intra‐cultivar variations masking this effect, only to be revealed when both intra‐ and inter‐cultivar variations are considered. This observation further highlights the need to consider multiple perspectives when analyzing complex transcriptomic patterns.

### 4.3 Tissue‐specific gene expression of two local olive tree cultivars

**FIGURE 2:**
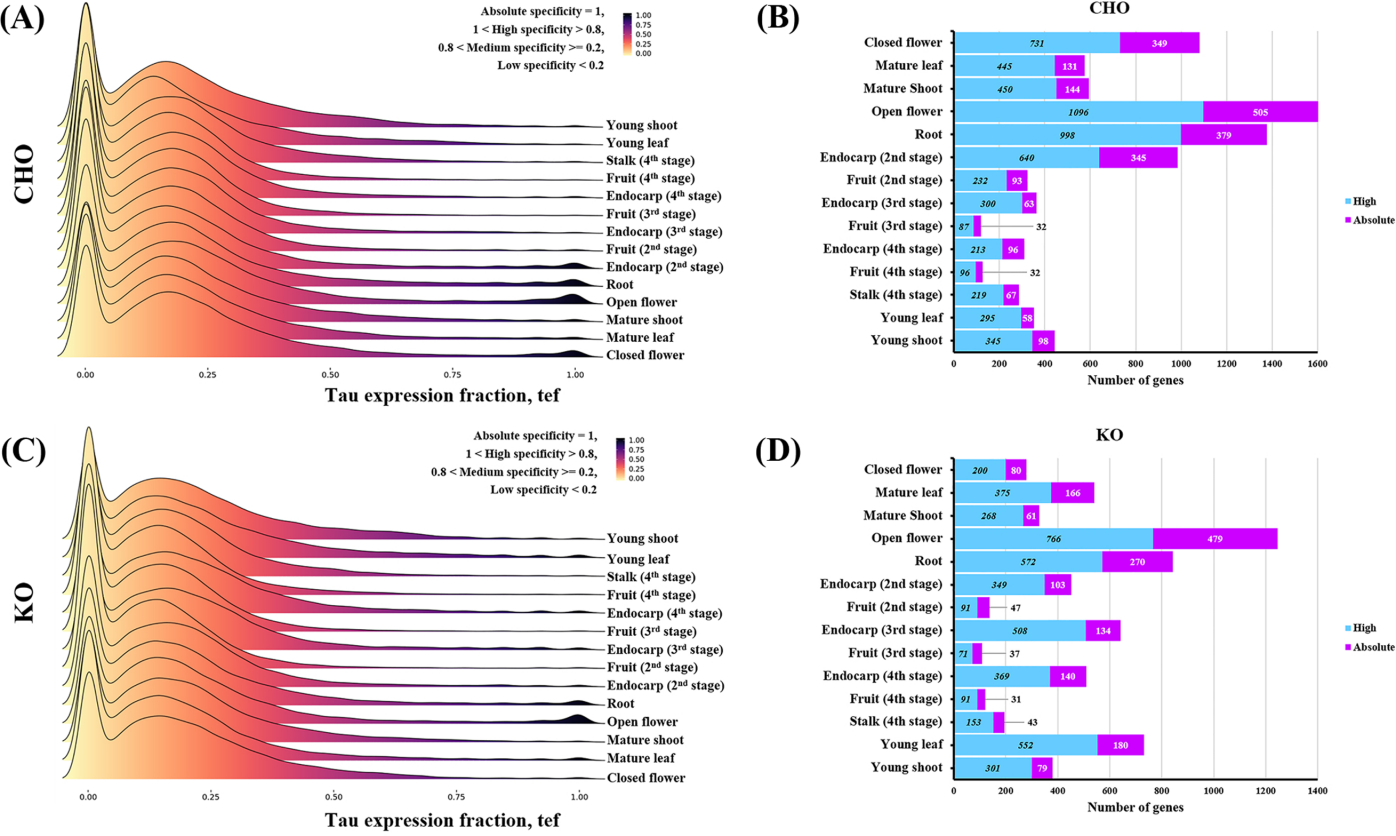
Tissue‐specific gene expression of two Greek olive tree cultivars. The figure shows tissue‐specific gene expression of CHO (A and B) and KO (C and D) tissue types and developmental stages. (A, C) Gene tissue‐specific expression as a function of *tef* values for 14 tissue types/ developmental stages. The color‐scale legend represents *tef* values, ranging from 0 (no specificity) to 1 (absolute specificity). (B, D) Barplots of the gene number per specificity level for 14 tissue types/ developmental stages.

Gene specificity was estimated for all tissue types and developmental stages using the *tau* and *tef* coefficients. Figure 2 shows gene specificity per tissue type and developmental stage of the two cultivars based on the *tef* value and the proportion of genes in each category. There was a significant variation in the number of tissue‐specific genes amongst samples, which covered only a small fraction of the total transcriptome, even in tissues with distinctively high levels of tissue‐specific gene expression, such as the root, the endocarp (stage 2), and the flowers of CHO (Figure 2A, B), as well as the open flower and root tissues of KO (Figure 2C, D). The bulk of the transcriptome consisted mainly of housekeeping genes, followed by genes with moderate specificity (Figure 2 and Supplementary Data S3). Amongst the CHO tissues with the highest number of tissue‐specific genes were the open flower, the root, the closed flower, and the stage 2 endocarp (Supplementary Data S3). For the KO cultivar, the tissues with the highest values of tissue‐specific genes were the open flower, the root, the young leaf, and the Stage 3 endocarp (Supplementary Data S3).

### 4.4 Differential gene expression amongst tissues in local olive tree cultivars

**FIGURE 3:**
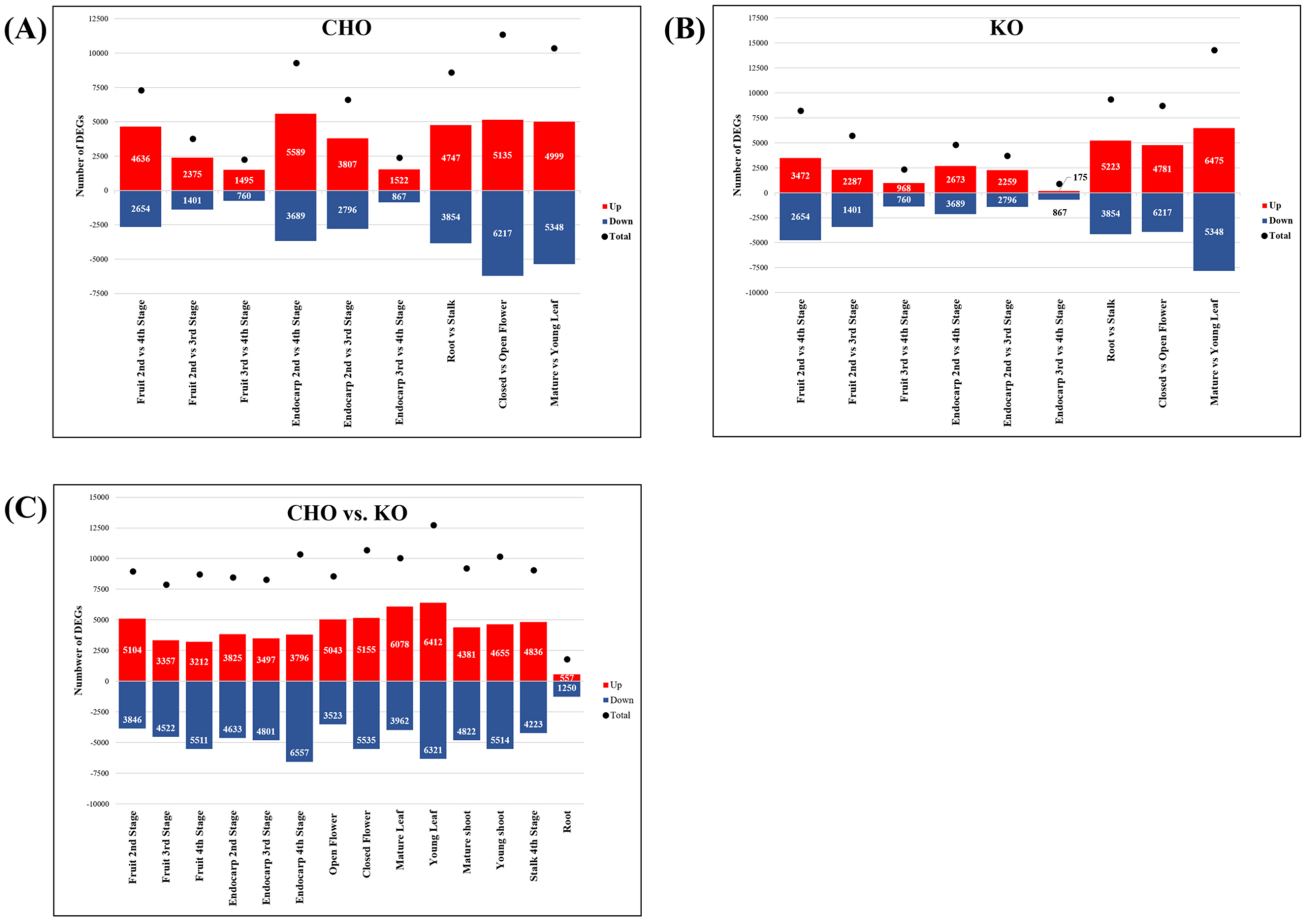
Number of differentially expressed genes (DEGs) amongst the CHO and KO cultivars. The barplots depict the number of up‐regulated (red) and down‐regulated (blue) DEGs amongst CHO tissues (A), KO tissues (B), as well as CHO vs. KO tissue comparisons (C). Dots represent the total number of DEGs for each comparison. The DEG datasets were filtered before counting for statistical significance (*p*_*adj*_ ≤0.05) and Log_2_ Fold Change (−1 < FC <1).

**FIGURE 4:**
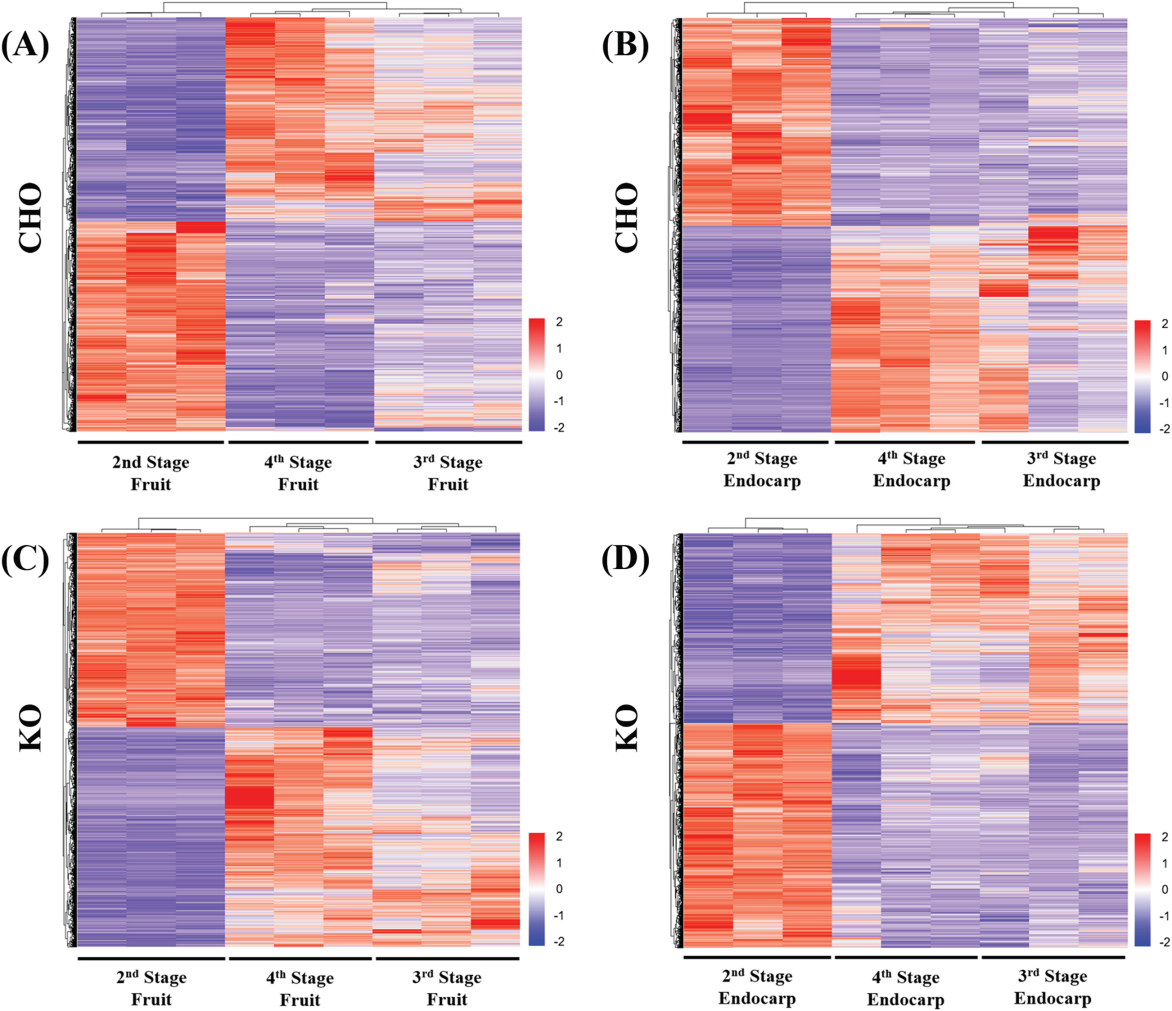
Gene expression patterns of fruit and endocarp developmental stages. Hierarchical clustering heatmaps of the fruit (A and C) and endocarp (B and D) tissues for the CHO (A and B) and KO (C and D) transcriptomes. The triplicates are shown independently for each tissue type/ developmental stage per cultivar. The color‐scale legend represents Log_2_‐ transformed TPM values.

To get an insight into the tissue‐ and genotype‐specific mechanisms that underlie the development and function of the studied olive cultivars, the transcriptomes of several tissue types and developmental stages were compared within and between cultivars to identify differentially expressed genes (DEGs). Regarding the CHO cultivar, both closed vs. open flowers and mature vs. young leaves showed the highest total numbers of DEGs (>10 000), indicating high variation in gene expression amongst these tissues (Figure 3A and Supplementary Data S4). Although all the fruit and endocarp comparisons in this cultivar exhibited lower total numbers of DEGs, the young vs. mature stages (stage 2 vs. stage 4) for both tissue types showed higher total numbers of DEGs than stage 2 vs. 3 and Stage 3 vs. 4 comparisons (Figure 3A). These highly contrasting gene expression patterns between young (stage 2) and mature (stage 4) developmental stages are also reflected by the gene expression heatmaps, which showed highly complementary expression profiles between stage 2 and stage 4, while an intermediate profile for Stage 3 tissues (Figure 4A, B). This disparity between young and mature endocarp and fruit tissues has also been observed in the combinatorial PCA analysis of both the CHO and KO samples (Supplementary Figure 1), further supporting the presence of characteristic transcriptional differences amongst different ontogenetic stages in both cultivars.

In the KO cultivar, the young vs. mature leaves showed the highest total number of DEGs (>12 500), followed by similar numbers for the root vs. stalk and closed vs. open flower comparisons (Figure 3B, Supplementary Data S4). The DEG comparisons of the fruit and endocarp tissues showed a profile similar to the one observed in CHO tissues (Figure 3A, B), with the early developmental stage 2 exhibiting highly variable gene expressions compared to that of the more mature tissues (stage 4). In contrast, Stage 3 tissues had an intermediate gene expression in both stages (Figure 4C, D, Supplementary Data S4). Nevertheless, it is noteworthy that for the majority of the KO tissue comparisons, the total number of DEGs is lower than those of CHO tissues (Figures 3A, B), indicating higher levels of gene expression uniformity between KO tissues.

To unravel cultivar‐specific mechanisms, DEGs were also identified by comparing the transcriptomes across all analyzed tissues between the studied cultivars (CHO vs. KO; Figure 3C, Supplementary File 1, and Supplementary Data S4). More specifically, the young leaves showed the highest number of DEGs (>12 500) between the two olive cultivars, followed by the young shoot, the closed flowers, and the stage 4 endocarp tissues (>10000; Figure 3C). It is noteworthy that the DEG numbers remain relatively constant amongst the various fruit and endocarp stages, except for stage 4 endocarp, which showed a considerable increase in the number of down‐regulated DEGs in CHO (Figure 3C and Supplementary File 1). As such, any varietal differences are likely attributed to the biological processes in these tissues, especially for the young leaf and closed flower tissues, which showed the highest number of DEGs (Figure 3 and Supplementary Data S4).

To get an insight into the biological functions present in the differentially expressed genes from these tissues, GO enrichment analysis was performed, revealing that the young leaf tissue DEGs were significantly enriched in protein and carbohydrate metabolic processes, as well as photosynthesis‐related processes (Supplementary Table 3 and Data S5). This indicates that the young leaves between cultivars may differ in their capacity for photosynthesis and protein synthesis. The closed flower DEGs showed significant enrichment in microtubule‐based processes (Supplementary Table 3 and Data S5). Microtubule isotropy has been associated with flower meristem function and morphology (Abad et al., 2017). Thus, it can be extrapolated that developmental program differences in microtubule genes between the two olive cultivars may lead to differences in flower development.

Notably, the root tissues showed the lowest number of DEGs (Figure 3C and Supplementary Data S4), which suggests functional conservation in root gene expression programs between cultivars. Conservation of root developmental programs was also observed amongst diverse vascular plant species, even in the lycophyte *Selaginella moellendorffii* Hieron., which has a distinct root morphology (Huang & Schiefelbein, 2015). It has also been hypothesized that roots have independently evolved at least twice in the evolutionary history of plants (Shekhar et al., 2019). Hence, it is apparent that root development and function require a level of stereotypy even among diverse plant lineages, which might explain the strict commonality in gene expression observed in the CHO and KO root transcriptomes. The GO enrichment analysis for the DEGs datasets for all tissue comparisons is described in detail in Supplementary File 1.

### 4.5 Gene expression trends in fruit and endocarp tissues

**FIGURE 5:**
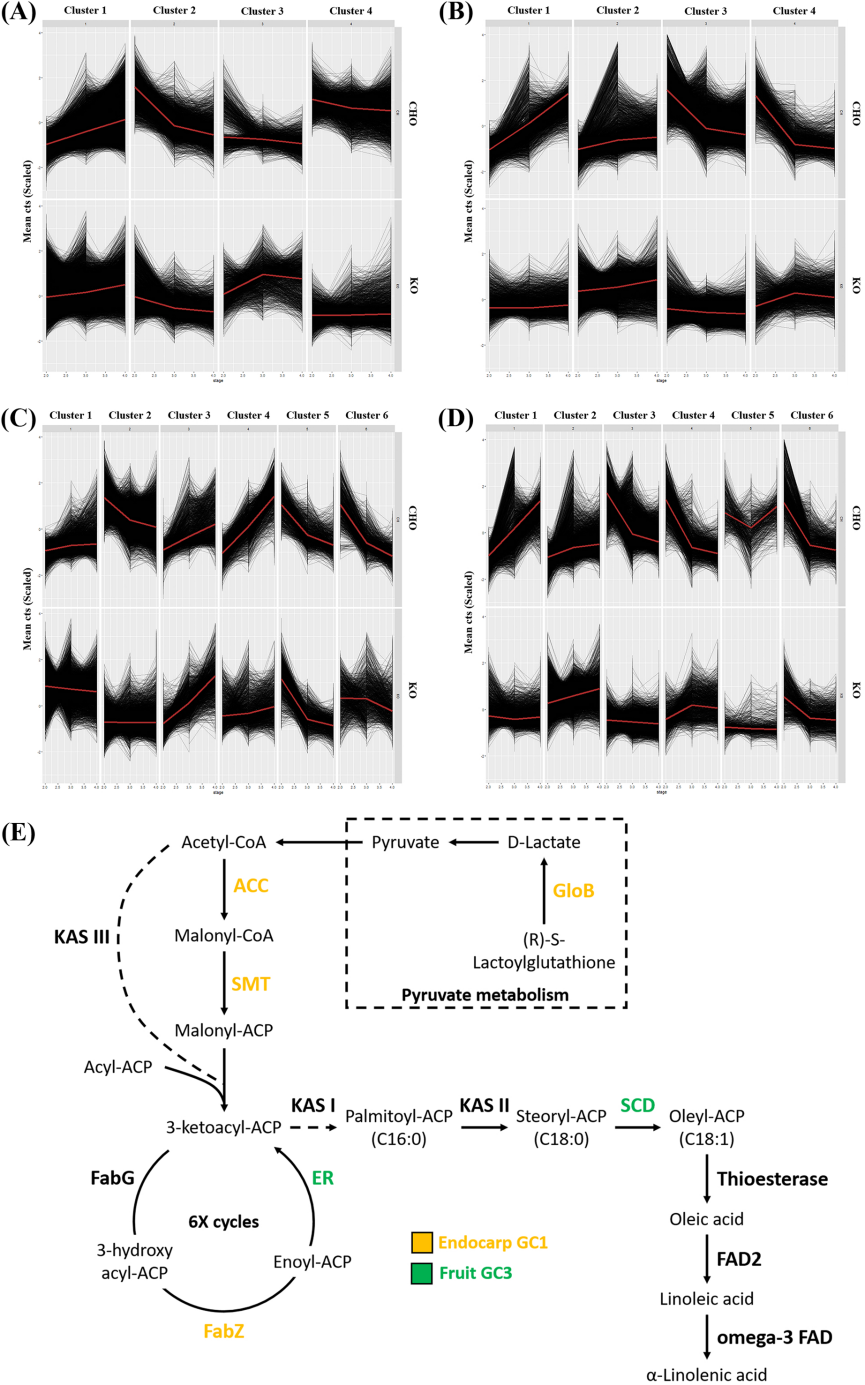
Gene expression patterns of the CHO and KO co‐expression modules. The 4‐cluster (A and B) and 6‐cluster (C and D) analyses are shown here. Each cluster depicts the expression trends of co‐expressed genes between the two cultivars across the three developmental stages (stages 2–4) for the fruit (A and C) and the endocarp (B and D) transcriptomes. (E) Graphical depiction of the oleic acid biosynthesis pathway in olive. The most highly significant CHO and KO co‐expressed fatty acid biosynthesis genes are color highlighted for the endocarp GC1 (orange) and the fruit GC3 (green) of the 4‐cluster analysis, which showed high enrichment in fatty acid metabolism genes. Abbreviations: ACC, acetyl‐CoA carboxylase; ER, enoyl‐ACP reductase; FabG, β‐ketoacyl‐ACP reductase; FabZ, β‐hydroxyacyl‐ACP dehydrase; FAD, fatty‐acid desaturase; GloB, hydroxyacylglutathione hydrolase; KAS, β‐ketoacyl‐ACP synthase; SCD, stearoyl‐ACP desaturase; SMT, S‐malonyltransferase.

Given the importance of fruit‐related tissues affecting oil production and fruit properties for tabletop use, the transcriptome data of the fruit and endocarp tissues was further analyzed by performing a hierarchical clustering analysis of co‐expressed genes between the two cultivars (Figure 5). Assessment of the fruit and endocarp gene co‐expression modules of the 4‐cluster analysis revealed relatively homogeneous gene expression trends between the two cultivars, albeit with some notable variations in some of the gene clusters (Figure 5A, B, and Supplementary Data S6). For the fruit stages, slight variations were observed in GC2 and GC3 between cultivars (Figure 5A). GO enrichment analysis of these clusters revealed significant enrichment in genes involved in protein repair, protein phosphorylation, cell‐wall structure, and photosynthesis, which are significantly differentially expressed with the onset of fruit development between the two cultivars (Supplementary Table 4 and Data S7). In contrast, gene clustering for the endocarp tissues revealed less homogenous patterns, with GC1, GC3, and GC4 exhibiting contrasting trends between cultivars (Figure 5B). GO enrichment analysis of the endocarp GC1, which contains genes that are up‐regulated with endocarp maturation in CHO (Figure 5B), showed highly significant enrichments in protein synthesis‐related processes (Supplementary Table 4 and Data S7). In contrast, the genes in GC3, which exhibit the opposite pattern (Figure 5B), showed significant enrichments in lipid metabolism, positive regulation of transcription, and microtubule‐based processes (Supplementary Table 4 and Data S7).

A 6‐cluster analysis was also performed for the CHO/KO co‐expressed genes for both the fruit and endocarp tissues (Figure 5C, D, and Supplementary Data S6). Regarding the fruit tissues, there was a comparable expression of genes in the various gene clusters with the onset of fruit development for both cultivars, except for the GC2 and GC4 genes (Figure 5C). More specifically, the GC2 genes of the 6‐cluster analysis showed a significantly gradual decreased expression in CHO compared to KO, while genes in GC4 showed the inverse pattern (Figure 5C). Functional enrichment analysis of the fruit GC2 revealed significant enrichment in defense responses and phosphorus metabolism. In contrast, the fruit GC4 exhibited significant enrichment in amide metabolic processes and protein and nucleotide‐related biosynthetic processes (Supplementary Table 5 and Data S7). Concerning the 6‐cluster analysis of the endocarp tissues, significant differences in gene expression trends between cultivars were observed for the GC1, GC3, and GC4 clusters (Figure 5D). GO enrichment analysis of the endocarp GC1 revealed significant enrichment in genes involved in protein synthesis‐related processes and monosaccharide metabolism, GC3 showed significant enrichment in chromatin assembly and RNA silencing mechanisms, while GC4 genes are involved in protein phosphorylation‐related processes (Supplementary Table 5 and Data S7). The GO enrichment analysis for the before‐mentioned gene clusters is detailed in Supplementary File 1.

### 4.6 Detection of genes involved in oil production and fruit shape and size

#### 4.6.1 Highly specific genes involved in oil production and fruit development

To gain insights into the genetic mechanisms underlying olive oil production and olive fruit development between cultivars, the presence of known fruit size and shape and oil production‐related genes (Supplementary Data S9 and S10) were analyzed in the CHO and KO highly specific genes datasets (*tau* >0.8; Supplementary Data S3). Regarding oil production, the highly‐specific gene datasets revealed similar genetic processes in both cultivars (Table 1 and Supplementary Data S9). Amongst these genes, an acyl‐CoA oxidase (Oeu015636.1) and a malate dehydrogenase (Oeu063213.1) showed absolute expression in the CHO open and closed flowers, respectively (Table 1 and Supplementary Data S9). Furthermore, a malate dehydrogenase gene (Oeu053173.1) was also identified to have absolute specificity in the CHO mature leaf (Table 1 and Supplementary Data S9). In contrast to CHO, known oil‐production genes also showed absolute specificity in the KO fruit stage 2, apart from the flower and mature leaf tissues (Table 1 and Supplementary Data S9). Detection of fatty acid metabolism genes in the flower tissues in both cultivars indicates that fatty acid biosynthesis is likely to initiate even before the onset of fruit development.

**TABLE 1:** Identification of known oil production and fruit size/shape‐related genes with absolute tissue specificity (*tau* = 1) in CHO and KO. The complete list of genes with high specificity (*tau* >0.8) can be found in Supplementary  Data S9 and S10.

Process	Cultivar	Gene ID	Tissue type	Description
Oil production	CHO	Oeu015635.1	Open flower	acyl‐CoA oxidase
		Oeu015636.1	Open flower	acyl‐CoA oxidase
		Oeu063213.1	Closed flower	malate dehydrogenase
		Oeu053173.1	Mature leaf	malate dehydrogenase
		Oeu042873.1	Root	acyl‐CoA oxidase
	KO	Oeu024466.1	Open flower	acyl‐[acyl‐carrier‐protein] desaturase
		Oeu032133.1	Open flower	acyl‐CoA oxidase
		Oeu011196.1	Mature leaf	fatty acyl‐CoA reductase
		Oeu015633.1	Fruit (2nd stage)	acyl‐CoA oxidase
		Oeu015636.1	Root	acyl‐CoA oxidase
Fruit size/ shape development	CHO	Oeu010545.1	Closed flower	MADS‐box transcription factor
		Oeu052267.1	Closed flower	MADS‐box transcription factor
		Oeu061769.1	Closed flower	MADS‐box transcription factor
		Oeu043454.1	Endocarp (2nd stage)	MADS‐box transcription factor
		Oeu064521.1	Endocarp (2nd stage)	MADS‐box transcription factor
		Oeu017585.1	Mature leaf	MADS‐box transcription factor
		Oeu016647.1	Open flower	MADS‐box transcription factor
		Oeu036693.1	Open flower	homeobox‐leucine zipper protein
		Oeu009933.1	Root	MADS‐box transcription enhancer factor 2A
		Oeu042086.1	Root	histidine‐containing phosphotransfer protein
		Oeu064601.1	Root	MADS‐box transcription factor
		Oeu026782.1	Stalk	histidine‐containing phosphotransfer protein
	KO	Oeu064823.1	Closed flower	phytochrome‐interacting factor 3
		Oeu051415.1	Endocarp (2nd stage)	MADS‐box transcription factor
		Oeu061769.1	Endocarp (2nd stage)	MADS‐box transcription factor
		Oeu010457.1	Endocarp (4th stage)	MADS‐box transcription factor
		Oeu009929.1	Open flower	MADS‐box transcription factor
		Oeu016647.1	Open flower	MADS‐box transcription factor
		Oeu020126.1	Open flower	homeobox‐leucine zipper protein
		Oeu041903.1	Open flower	homeobox‐leucine zipper protein
		Oeu051193.1	Open flower	MADS‐box transcription factor
		Oeu053261.1	Open flower	MADS‐box transcription factor
		Oeu059535.1	Open flower	homeobox‐leucine zipper protein
		Oeu024642.1	Root	MADS‐box transcription factor
		Oeu024986.1	Root	MADS‐box transcription factor
		Oeu043454.1	Young leaf	MADS‐box transcription factor
		Oeu052267.1	Young shoot	MADS‐box transcription factor

Apart from oil‐production genes, we were also interested in genes known to play a role in fruit development, which directly affect drupe quality and may also influence oil production. Screening for fruit size‐ and shape‐related genes in the highly specific gene datasets for both cultivars revealed the presence of *homeobox‐leucine zipper* and *MADS*‐box transcription factor genes (Table 1 and Supplementary Data S10). *SHATTERPROOF*‐like MADS‐box transcription factors showed absolute specificity in CHO root, closed and open flower, stage 2 endocarp, and mature leaf tissues (Table 1 and Supplementary Data S10). In KO, MADS‐box transcription factors showed absolute specificity in open flower, root, stage 2 and stage 4 endocarp, as well as young leaf and shoot tissues (Table 1 and Supplementary Data S10). MADS‐box transcription factors were shown to control fruit dehiscence and gynoecium development in Arabidopsis (Liljegren et al., 2000; Colombo et al., 2010). Although fruit dehiscence is not a process that occurs in olives, these transcription factors may have a broader role in developing the flower and endocarp tissues. Interestingly, *SHATTERPROOF*‐like MADS‐box transcription factors were also detected in the root of both cultivars (Table 1 and Supplementary Data S10), which is not an unexpected result, given that MADS‐box genes were also shown to control nutrient‐induced changes in the Arabidopsis root (Zhang & Forde, 1998). Regarding homeobox transcription factors, both cultivars showed absolute specificity of *WUS* (*WUSCHEL*)‐like homeobox genes in the open flower tissue (Table 1 and Supplementary Data S10). *WUS* was shown to activate floral patterning in Arabidopsis (Ikeda et al., 2009), hence these homeobox genes may also play a similar role in olive tree flower development.

It is noteworthy that *histidine‐containing phosphotransferase* (*HPT*) genes showed absolute specificity only in CHO stalk (Oeu026782.1) and root (Oeu042086.1) tissues, while a *phytochrome interacting factor 3* (Oeu064823.1) showed absolute specificity only in KO closed flower (Table 1 and Supplementary Data S10). The *HPT* genes are involved in the histidine‐to‐aspartate phosphorelay signal transduction systems responsible for the propagation of environmental stimuli via phytohormone activity (Suzuki et al., 2000). Thus, such signal transduction mechanisms are likely to play a more essential role in the development of CHO. Contrarily, the exclusive detection of a *phytochrome interacting factor 3* in the KO flowers, which is involved in the hormonal regulation of seed dormancy (Oh et al., 2009), suggests that such regulatory mechanisms may have a more prominent role in KO flower development.

#### 4.6.2 Differentially expressed genes

**FIGURE 6:**
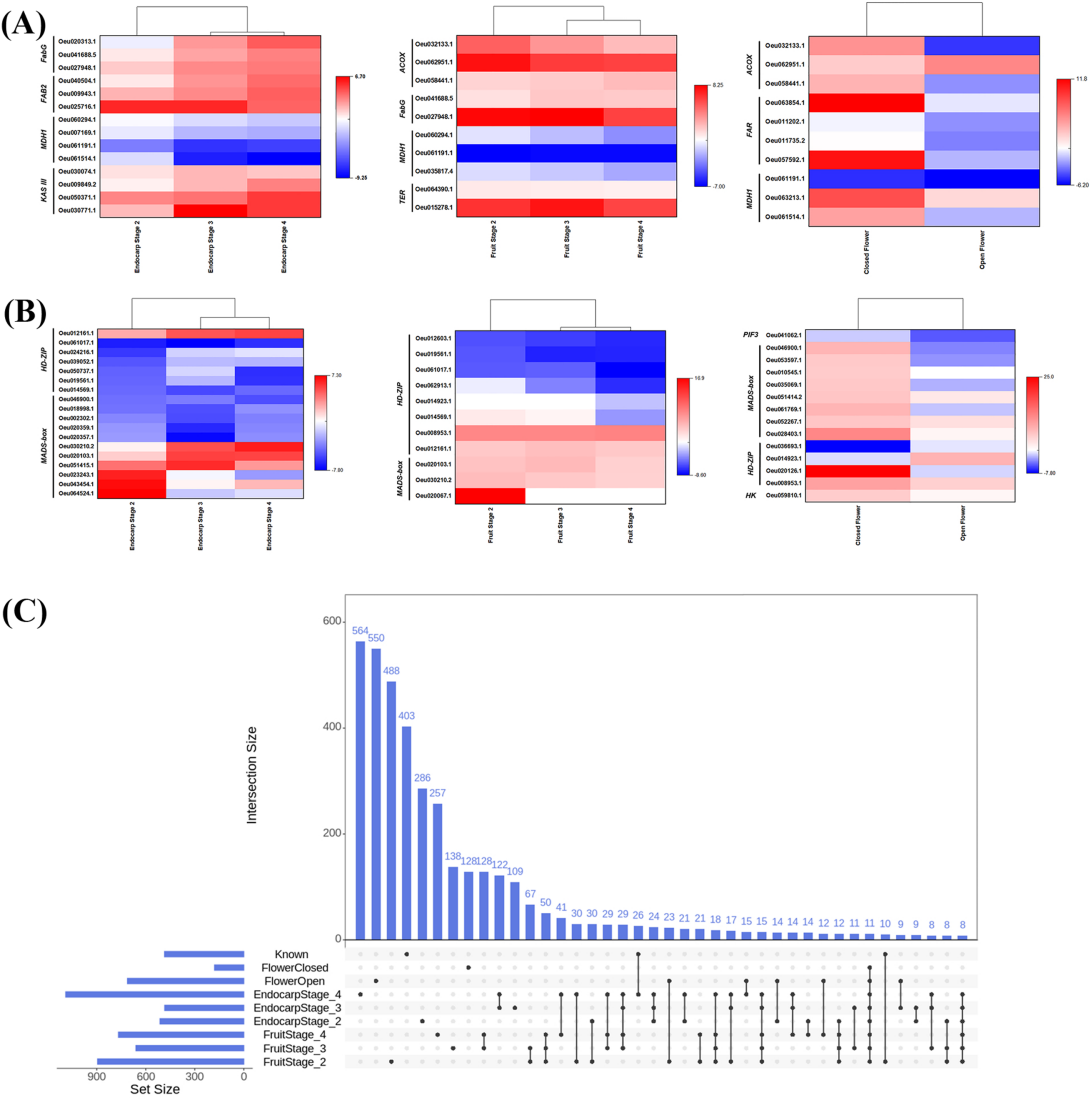
Identification of genes reported to be involved in oil production and fruit shape/ size development. (A and B) Hierarchical clustering heatmaps of the most significant and highly expressed CHO vs. KO DEGs known to be involved in fatty acid metabolism (A) and fruit development (B). The color‐scale legend represents Log_2_ Fold Change (FC) values, with red (Log_2_ FC >0) indicating higher CHO and blue (Log_2_ FC <0) higher KO expression. Abbreviations: ACOX, acyl‐CoA oxidase; FAB2, acyl‐[acyl‐carrier‐protein] desaturase; FabG, 3‐oxoacyl‐[acyl‐carrier protein] reductase; FAR, fatty acyl‐CoA reductase; HD‐ZIP, homeobox‐leucine zipper; HK, hexokinase; KASIII, 3‐oxoacyl‐[acyl‐carrier‐protein] synthase III; MADS‐box, MADS‐box transcription factor; MDH1, malate dehydrogenase; PIF3, phytochrome‐interacting factor 3; and TER, enoyl reductase. (C) Intersection graph of CHO vs. KO DEGs amongst flower, endocarp, and fruit tissues. The “Known” gene set represents genes with documented function in oil production‐related processes and fruit size/shape development. Black colored dots indicate the presence of the corresponding intersection set in each respective tissue type and developmental stage. Lines indicate intersections amongst tissues. The original DEGs data (Supplementary Data S4) was further filtered based on the p_adj_ value (*p*_*Cutoff*_ ≤ 10e^−16^).

The CHO vs. KO fruit, endocarp, and flower DEG lists were also screened for the presence of known oil production‐related genes (Supplementary Data S9). During endocarp maturation (stages 3 and 4) the CHO tissues showed significant differential expression of 3‐oxoacyl‐[acyl‐carrier protein] synthase III, 3‐oxoacyl‐[acyl‐carrier protein] reductase, and acyl‐[acyl‐carrier‐protein] desaturase DEGs, while in the KO mature endocarp tissues, malate dehydrogenase DEGs were the most significant and highly expressed (Figure 6A and Supplementary Data S9). In the fruit DEGs dataset, acyl‐CoA oxidase, 3‐oxoacyl‐[acyl‐carrier protein] reductase, and enoyl reductase DEGs were significantly increased in CHO, while malate dehydrogenase DEGs were significantly upregulated in KO (Figure 6A and Supplementary Data S9). In contrast to the endocarp tissues, differential gene expression was relatively constant for most of the highly expressed DEGs throughout the onset of fruit development (Figure 6A and Supplementary Data S9). Finally, in the flower tissues, both cultivars showed significantly high expression of acyl‐CoA oxidase, fatty acyl‐CoA, reductase, and malate dehydrogenase DEGs (Figure 6A and Supplementary Data S9). Although fatty acyl‐CoA reductase and malate dehydrogenase DEG expression was reduced in the open flowers of CHO, expression in KO open flowers increased (Figure 6A and Supplementary Data S9). It is noteworthy that the most significant and highly expressed acyl‐CoA oxidase DEGs exhibited higher expression in the CHO closed flowers, except for Oeu062951.1, while in KO, these genes were upregulated in the open flower tissue (Figure 6A and Supplementary Data S9). Overall, it becomes apparent that for the most significant olive tree tissues for oil production, there is a consistent requirement for malate dehydrogenase activity throughout development in KO, while in CHO, oil production‐related gene functions are tissue‐type and developmental‐stage dependent.

Assaying the endocarp, fruit, and flower CHO vs. KO DEGs datasets for genes that are involved in fruit development revealed that most MADS‐box transcription factors showed the highest significant expression in CHO tissues. In contrast, *WUS*‐like homeobox transcription factors showed a significant fold change increase in the corresponding KO tissues (Figure 6B and Supplementary Data S10). This was remarkably prominent in the endocarp and fruit stages. This finding indicates a contrasting role of the MADS‐box and homeobox transcription factors in the studied cultivars. MADS‐box genes play an important role in flower and fruit development in many plant species, while downregulation of these genes leads to several flower, seed, and fruit defects, including fruit size reduction (Ng & Yanofsky, 2001; Seymour et al., 2011; Ireland et al., 2013; Guo et al., 2022). Given that “Chondrolia Chalkidikis” is characterized by fleshier and larger fruits than “Koroneiki”, this may explain why MADS‐box genes show more prominent expression in this cultivar.

#### 4.6.3 CHO/KO co‐expressed gene modules

Known oil production and fruit shape/size‐related genes (Supplementary S9 and S10) were also surveyed in the fruit and endocarp gene cluster datasets of the CHO/KO co‐expression modules (Supplementary Data S6). Gene cluster selection was carried out regarding profile variability in gene co‐expression trends between cultivars and their enrichment in genes involved in the corresponding processes. In terms of oil production, the fruit GC3 and endocarp GC1 of the 4‐cluster analysis were selected (Figure 5 and Supplementary Data S8). Apart from the common fatty acid biosynthesis genes in both tissues, several genes showed tissue specificity, including stearoyl‐CoA desaturase (delta‐9 desaturase) and enoyl reductase genes that were present only in the fruit GC3, while s‐malonyltransferase, acetyl‐CoA carboxylase, hydroxyacylglutathione hydrolase, and 3‐hydroxy acyl‐CoA dehydratase genes being present only in the endocarp GC1 (Figure 5E, Table 2 and Supplementary Data S6). Notably, a hydroxy fatty acyl‐CoA dehydratase PASTICCINO2 in *A. thaliana* was shown to play an essential role in several aspects of plant development, including embryo development, leaf shape, and size, as well as epidermal cell morphology (Bach et al., 2008). Thus, fatty acid biosynthetic genes are not only essential for oil production but might play a more prominent role in the function of the plant tissue as a whole.

**TABLE 2:** Unique KEGG identifiers of indicative CHO/KO co‐expression gene clusters. The gene clusters were selected based on differential expression patterns between cultivars and their significant enrichment in oil production‐ and fruit development‐related genes.

Process	Tissue	Cluster	KEGG	Blast description
Oil production	Fruit	GC3 (4‐cluster analysis)	K00026	malate dehydrogenase
			K00232	acyl‐CoA oxidase
			K00507	stearoyl‐CoA desaturase (delta‐9 desaturase)
			K00666	fatty‐acyl‐CoA synthase
			K01895	acetyl‐CoA synthetase
			K01897	long‐chain acyl‐CoA synthetase
			K09458	3‐oxoacyl‐[acyl‐carrier‐protein] synthase II
			K10258	enoyl reductase
	Endocarp	GC1 (4‐cluster analysis)	K00025	malate dehydrogenase
			K00232	acyl‐CoA oxidase
			K00645	[acyl‐carrier‐protein] S‐malonyltransferase
			K00648	3‐oxoacyl‐[acyl‐carrier‐protein] synthase
			K00666	fatty‐acyl‐CoA synthase
			K01069	hydroxyacylglutathione hydrolase
			K01895	acetyl‐CoA synthetase
			K01897	long‐chain acyl‐CoA synthetase
			K01962	acetyl‐CoA carboxylase carboxyl transferase subunit alpha
			K02160	acetyl‐CoA carboxylase biotin carboxyl carrier protein
			K10703	3‐hydroxy acyl‐CoA dehydratase
Fruit size/ shape development	Fruit	GC2 (6‐cluster analysis)	K00818	acetylornithine aminotransferase
			K00844	hexokinase
			K03500	ribosomal RNA small subunit methyltransferase B
			K09264	MADS‐box transcription factor
			K09338	homeobox‐leucine zipper protein
			K09349	homeobox protein Nkx‐5
			K09833	homogenitisate phytyltransferase
			K12126	phytochrome‐interacting factor 3
			K14490	histidine‐containing phosphotransfer protein
	Endocarp	GC3 (6‐cluster analysis)	K00457	4‐hydroxyphenylpyruvate dioxygenase
			K00844	hexokinase
			K00884	N‐acetylglucosamine kinase
			K05928	tocopherol O‐methyltransferase
			K09264	MADS‐box transcription factor
			K09265	MADS‐box transcription factor
			K09338	homeobox‐leucine zipper protein
			K09349	homeobox protein Nkx‐5
			K09833	homogenitisate phytyltransferase
			K09834	tocopherol cyclase

Regarding fruit shape and size development, the fruit GC2 of the 6‐cluster analysis and the endocarp GC3 were selected (Figure 5C‐D and Supplementary Data S8). The genes in these clusters showed a maturation‐dependent down‐regulation in CHO tissues, while in KO, they retained a relatively constant expression across all developmental stages in both tissues (Figure 5C and D). Apart from MADS‐box and homeobox‐containing transcription factors, genes with no direct association with developmental processes were also identified in these clusters, including hexokinases and tocopherol production genes (Table 2 and Supplementary Data S6). Hexokinases are involved in plant sugar signal transduction, subsequently regulating several plant developmental processes (Xiao et al., 2000). On the other hand, tocopherol‐producing proteins, such as tocopherol cyclases and homogentisate phytyltransferases, are involved in producing vitamin E (Collakova & DellaPenna, 2003; Sattler et al., 2003). As such, it is amenable to speculate that fruit and endocarp developmental processes, sugar signaling, and vitamin E production may have different temporal frames between the two tested Greek olive cultivars.

#### 4.6.4 Novel genes involved in oil production and fruit development

To identify novel genes that differentiate the two olive cultivars in terms of oil production and fruit size, the CHO vs. KO flower, endocarp, and fruit DEGs datasets (Supplementary Data S4) were further filtered for the most significant DEGs (*p*_*Cutoff*_ ≤ 10e‐16) and the generated lists were compared for gene intersections (Supplementary Data S11). Amongst the various gene sets, the intersections with the highest number of highly significant DEGs were only detected either in the open flower (inter. FlowerOpen; 550 DEGs), stage 2 (inter. EndocarpStage_2; 286 DEGs), and stage 4 endocarp (inter. EndocarpStage_4; 564 DEGs), or in stage 2 (inter. FruitStage_2; 488 DEGs) and stage 4 fruits (inter. FruitStage_4; 257 DEGs), indicating that a large number of the highly significant DEGs are tissue specific (Figure 6C and Supplementary Data S11). Two of the medium‐sized intersections were detected in stages 3 and 4 endocarp (inter. EndocarpStage_3–4; 122 DEGs), as well as Stage 3 and 4 fruit tissues (inter. FruitStage_3–4; 128 DEGs), indicating a putative involvement of these DEGs in the maturation processes (Figure 6C and Supplementary Data S11). The rest of the intersections comprised of a small number of DEGs, including the intersections with the list of genes that have been reported to play a role in oil production and fruit development (inter. Known/ EndocarpStage_4; 26 DEGs and Known/ FruitStage_2; 10 DEGs). It is noteworthy that a large number of genes with documented function in oil production and fruit development (inter. Known; 403 DEGs) are not present in the tested CHO vs. KO DEGs datasets (Figure 6C and Supplementary Data S11), indicating that most of these genes may have common expressions in fruit, endocarp, and flower tissues amongst cultivars.

### 4.7 Database development

The generated raw transcriptome and processed data for all tissue types per cultivar were made available via the public database Sequence Read Archive (SRA; BioProject: PRJNA763324) and the in‐house developed database GrOlivedb (www.GrOlivedb.com). The GrOlivedb is a web‐based, curated, relational database developed using Tripal v3 (Spoor et al., 2019) that builds upon the open‐source Drupal content management system and the GMOD Chado database schema (Mungall et al., 2007). GrOlivedb hosts datasets consisting of metadata, annotated tables of co‐expressed gene modules, comparative gene expression profiles, and tissue‐based gene specificity data from analyzing 14 different tissue types and developmental stages for both cultivars. Additionally, the GrOlivedb database provides analysis and visualization tools for nucleotide sequence‐based search and gene code‐based search, facilitating direct access to our processed data and enabling additional independent research.

The variations in gene expression between the KO cultivar, primarily used for oil production, and CHO, primarily used for table olive production, can be used as a foundation for genetic‐assisted breeding investigations. Aiming to assist such future efforts, GrOlivedb offers two comprehensive search and visualization tools utilizing the transcript sequences and the normalized gene counts. Those tools depend upon the Tripal extension modules NCBI BLASTn (Altschul et al., 1997) and Expression (https://github.com/tripal/tripal_analysis_expression.git). The NCBI BLASTn tool can execute sequence similarity searches within the Koroneiki and the Chondrolia Chalkidikis consensus sequences while providing sequence similarity visual aids. The consensus sequences used to build the BLASTn databases were derived by the SamTools (v. 1.16) integrated Bayesian “Gap5” consensus algorithm (Boulding et al., 1993), utilizing the transcriptomic heterozygous consensus sequence data of the mature leaf tissues of each cultivar and their mapping quality scores. In parallel, the Expression tool allows the visual representation of the level of expression of specific genes, given their gene code, between the tissues of each cultivar (Koroneiki, Chondrolia Chalkidikis), utilizing normalized gene count data for each tissue and developmental stage.

## 5 CONCLUSIONS

In conclusion, our analyses revealed tissue‐specific and differentially expressed genes between the CHO and KO cultivars that could be utilized for biomarker development and assisting future transcriptomic studies and applications. Furthermore, the current analysis revealed the differences in gene expression between a cultivar mainly used for oil production (“Koroneiki”) and one used primarily for table olives production (“Chondrolia Chalkidikis”). The GrOlivedb (www.GrOlivedb.com) database was created to access the transcriptomic data on all olive tissues and cultivars. The broad transcriptomic dataset presented herein will form the basis for future breeding studies and provide valuable resources to facilitate olive tree crop improvement. The current gene expression atlases will help with the application of functional genomics and molecular breeding in olive trees and offer a thorough understanding of global gene expression in the primary tissue types and developmental stages of olives.

## AUTHOR CONTRIBUTIONS

Conceptualization was done by A. M., I. G. and P. M. Data were curated by G. L., I. K., M. A., A. C. B., and D. V. The formal analysis was performed by G. L., I. K., A. C. B., and C. B. The investigation was done by G. L., I. K., G.‐M. N., A. C. B., C. S., M. Mi., and M. Ma.; Software responsibilities were on I. K. and M. I. and the methodology was on G. L., I. K., G.‐M. N., A. C. B., and C. B. Project administration was done by I. G. and P. M. and A. M., I. G. and P. M. were responsible for the resources. Supervision was given by I. G. and P. M. and funding acquisition was ensured by A. M., I. G. and P. M.; Visualization was done by G. L., I. K., C. B., and M. I. The writing of the original draft was performed by G. L. and I. K. and the writing at the review & editing process by G. L., I. K., A. M., A. C. B., A. K., I. M., G. K., C. B., I. G., D. K., and P. M. All authors reviewed and approved the final manuscript.

## CONFLICT OF INTEREST STATEMENT

The authors declare no conflict of interest.

## Supporting information


**Supplementary Data S1** **A.** Sequencing read counts and quality control of the transcriptomes generated for the 14 tissue types/ developmental stages of the CHO olive tree cultivar.

**Supplementary Data S2** **B.** Mapping information of the transcriptomes generated for the 14 tissue types/ developmental stages of the KO olive tree cultivar.

**Supplementary Data S3** **B.** Gene tissue specificity expressed in tau values for KO tissue types/ developmental stages.

**Supplementary Data S4.** DEGs between CHO and KO tissues, as well as between tissues within each cultivar. The Log2 fold change, as well as the p and padj values, are shown for each gene per comparison.

**Supplementary Data S5** **N.** Functional enrichment analysis of Chondorlia (CHO) vs. Koroneiki (KO) root DEGs.The GO terms are classified according to Molecular Function (MF), Biological Process (BP), and Cellular Component (CC).

**Supplementary Data S6** **D.** Chondrolia Chalkidikis (CHO) and Koroneiki (KO) endocarp co‐expression gene clusters (6‐cluster analysis).

**Supplementary Data S7** **J.** Functional enrichment analysis for the fruit GC4 genes of the CHO/KO 6‐cluster analysis.The GO terms are classified according to Molecular Function (MF), Biological Process (BP), and Cellular Component (CC).

**Supplementary Data S8.** Enrichment of CHO/KO co‐expression gene clusters in fruit shape/size and oil production‐related genes. The analysis was performed on gene clusters that exhibited differential gene expression trends between cultivars.

**Supplementary Data S9** **A.** “Known” olive oil production genes retrieved from annotation databases and related bibliography. This gene list was intersected with the CHO and KO highly‐specific gene dataset (Supplementary Data S3), as well as the CHOvsKO endocarp, fruit, and flower datasets (Supplementary Data S4) to identify oil production‐related genes.

**Supplementary Data S10** **A.** “Known” olive fruit shape and size genes retrieved from annotation databases and related bibliography. This gene list was intersected with the CHO and KO highly‐specific gene dataset (Supplementary Data S3), as well as the CHOvsKO endocarp, fruit, and flower datasets (Supplementary Data S4) to identify genes involved in fruit shape and size development.

**Supplementary Data S11** **C.** Chondrolia Chalkidikis (CHO) vs. Koroneiki (KO) Endocarp, Fruit, and Flower DEGs intersections with Known genes. The genes annotated as “Known” correspond to genes with documented function (based on bibliography) in fatty acid biosynthesis and/ or fruit development. Gene intersection coding follows the order of scoring in S11B.

**Data S1:** Supporting Information.

## Data Availability

The raw transcriptome data for both cultivars was published on GenBank with BioProject ID: PRJNA763324 (https://www.ncbi.nlm.nih.gov/bioproject/?term=Olea). The data that supports the findings of this study are available in the supplementary materials, which were submitted to the “Figshare” repository (https://doi.org/10.6084/m9.figshare.24994287). The GrOlivedb (www.GrOlivedb.com) database was created to access the annotated transcriptome of all the tissue types for both olive tree cultivars.
