# Supplementary material for: High‐resolution gene expression atlases of two contrasting major Greek olive (Olea europaea L.) tree cultivars for oil and table olive production
Source: Physiol Plant. 2024 Nov 5;176(6):e14600. doi: 10.1111/ppl.14600 (PMC11659808; doi:10.1111/ppl.14600)
Supplement: Supplementary file 12 — Data S1: Supporting Information. [file PPL-176-e14600-s007.docx]

**SUPPLEMENTARY MATERIAL**

**High-resolution gene expression atlases of two contrasting major Greek olive (*Olea europaea* L.) tree cultivars for oil and table olives production**

**Georgios Lagiotis^1^, Ioanna Karamichali^1^, Maria Astrinaki^2^, Androniki C. Bibi^2^, Despoina Vassou^2^, Georgia-Maria Nteve^1^, Anastasios Kollias^2^, Ioanna Manolikaki^3^, Christina Skodra^4^, Michail Michailidis^4^, Maria Manioudaki^4^, Marios Iakovidis^5^, Ioannis Ganopoulos^6,7^, Georgios Koubouris^3^, Athanassios Molassiotis^4^, Christos Bazakos^6,7,8^, Dimitris Kafetzopoulos^2,†^, and Panagiotis Madesis^1,9,*^**

**Materials and Methods**

**Plant material samplings and RNA extraction**

The plant material was collected from Greek olive tree cultivars that were morphologically and genetically characterized based on SSR and genomic data (Bazakos et al. 2023; Tourvas et al. 2023) as *Olea europaea* L. cv. “Chondrolia Chalkidikis” (CHO) and *O. europea* L. cv. “Koroneiki” (KO). The CHO samples were collected from trees grown at the Farm of the Aristotle University of Thessaloniki (Thessaloniki, Thermi, Greece) and the KO samples from trees at the National Olive Germplasm Bank of Greece at ELGO DIMITRA – Institute of Olive Tree, Subtropical Crops and Viticulture (Chania, Crete, Greece). Sampling per cultivar was performed from different locales, in order to capture the transcriptome of each cultivar grown in their native cultivation environment and to get information on their gene expression, especially in terms of oil production and fruit development that may underlie the variation observed in these characteristics in situ. Furthermore, these cultivars do not grow well in non-native and laboratory conditions. All samples were collected from 25-year-old trees in three biological replicates per organ, tissue type and developmental stage per cultivar (Supplementary Table 1). Organ developmental stage determination was carried out based on the annual growth stage approach, following the olive tree phenological stages (BBCH scale) described in Sanz-Cortés et al. 2002. Total RNA was extracted from 100 mg of fresh tissue material from each biological replicate per tissue type, using the Monarch Total RNA Miniprep Kit (NEB, Ipswich, MA, USA), according to the manufacturer’s instructions.

**RNA library construction and sequencing**

For RNA library construction, 2 μg of total RNA from three biological replicates per tissue type and cultivar were used. The RNA libraries were prepared using the NeBNext Ultra II Directional RNA Library Prep Kit for Illumina (NEB, Ipswich, MA, USA) and library indexing was performed using the ΝEBNext® Multiplex Oligos for Illumina (Set 1 and 2) kit (ΝΕΒ, Ipswich, MA, USA), according to the manufacturer’s instructions. Paired-end RNA library sequencing was performed using the NextSeq 550 system of Illumina (Illumina Inc., CA, USA). Paired-end sequencing was performed on the NextSeq500/550 platform (Illumina, San Diego, CA, USA), using the High Output Kit v2, 2x150 bp.

**Transcriptome mapping**

Raw next generation sequencing reads were filtered, removing low quality reads (cut-off value set to 20 for KO and 30 for CHO) and adaptor sequences, using Trim-Galore (V0.6.4; Babraham Bioinformatics - Trim Galore; <https://www.bioinformatics.babraham.ac.uk/projects/trim_galore/>). The clean reads were then mapped to the wild olive (*O. europaea* var. *sylvestris*) reference genome (Unver et al. 2017) using the mapper STAR V2.7.3a (Dobin et al. 2013). The counts of each gene were normalized using TPM (Transcripts Per Million) in order to focus only on significantly expressed genes.

Although new versions of the reference genome for the domesticated olive O. *europaea* var. *europaea* have been released (Jiménez-Ruiz et al. 2020; Julca et al. 2020; Rao et al. 2021), we used the wild variety genome, given test alignments of indicative CHO and KO tissues on the FARGA Oe9 domesticated olive genome showed high variation amongst the CHO and KO cultivars (Supplementary Table 2). More specifically, in CHO samples were consistently mapped slightly better on the wild olive reference than the common olive, while KO showed an average of 5% increase in the uniquely mapped reads percentage. Therefore, we opted in using the wild olive *O. europaea* var. *sylvestris* genome, where both cultivars perform the same (83% average uniquely mapped reads), which will allow for better comparisons between the two cultivars. Furthermore, the higher percentage of duplications in the FARGA Oe9 genome could significantly affect the gene counts observed. Another major point is that we have also employed the *O. europaea* var. *sylvestris* genome for our recently published work by (Bazakos et al. 2023), which will allow for complementarity between the transcriptomic data generated in this work with genes identified through GWAS and functional annotation validations, without the requirement for re-alignment of the genomic data. Additionally, chromosomal consensus sequences from the mature leaf samples of both the Koroneiki and the Chondrolia Chalkidikis transcripts were derived by the alignment (BAM) files and the mapping quality, using SamTools (v. 1.16) integrated Bayesian "Gap5" consensus algorithm (Boulding et al. 1993). Those consensus sequences were used to build the BLASTn databases published in the dedicated database GrOlivedb, as described below.

**Gene expression specificity analysis**

The mean values of the filtered gene raw counts were used to evaluate the expression specificity of the genes in the entirety of all grouped samples per tissue and cultivar, but also between the tissues of a specific cultivar, or between cultivars using the tau index of gene specificity (Yanai et al. 2005). The value of tau ranges from 0 to 1, indicating the level of specificity, as absolute specificity= 1, high specificity values between 0.8-1, medium specificity values between 0.2-0.8, and low specificity values< 0.2. The specificity of each gene for each individual tissue or cultivar was calculated using the tau expression fraction (*tef*). The R package Tispec (V0.99.0; <https://rdrr.io/github/roonysgalbi/tispec/>) was used for this analysis, while the R package ggplot2 (V3.3.2; <https://ggplot2.tidyverse.org/>.) was used for the visualization of the results.

**Differential gene expression analysis**

The differential analysis for the different tissues within a cultivar, as well as the tissues between cultivars, was performed using the DESeq2 (V1.32.0) R package (Love et al. 2014) on RStudio (https://www.rstudio.com/products/team/). Following the alignment of the obtained reads to the reference genome, the appropriate raw read count per gene table was constructed. The raw counts were then normalized using the estimateSizeFactors R function (Love et al. 2014), which utilizes the "median ratio method" described by Equation 5 in Anders and Huber study (Anders and Huber 2010). The normalization step also minimizes any differences between samples that might have occurred due to differences in sequencing depths. Following the differential analysis, the Enhanced Volcano R function (https://rdocumentation.org/packages/EnhancedVolcano/versions/1.11.3) was used to produce volcano plots of the results following the parameters *p_adj_*< 0.05, x axis= Log_2_ Fold Change (FC), and y axis= p-value (Supplementary Figure 2). The differentially expressed genes between the two cultivars were additionally filtered based on their *p_adj_* value (*p_Cutoff_* ≤ 10e-16) and compared to the genes that have been previously described to play a role in the oil production and the quality of the fruit (fruit size and shape). This comparison aimed at the identification of novel genes that may play a role in the oil production and fruit quality, while being differentially expressed between cultivars.

**Gene expression trends analysis with clustering**

Using the RNA-seq data from the fruit and endocarp tissues, the changes in the gene expression pattern were investigated, as follows: a) at different time points of development stages of the two tissues of a single cultivar, and b) at the same time point for each tissue between the two cultivars. The normalization of the raw read counts and the differential expression analysis were performed using the estimateSizeFactors R function and the DESeq2 (V1.32.0) R package (Love et al. 2014), respectively. Concerning the Differential Expression analysis, numeric contrast function (Love et al. 2014) was used in order to compare each condition against the average of the others. This was followed by the gene expression trends analysis in R using *p_adj_*< 0.01. The analysis revealed gene “populations” which exhibited similar expression trends across the developmental stages for the tissues, by utilizing the hierarchical clustering method (Reynolds et al. 2006). The parameters used were the Euclidean distance metric to calculate a “distance” metric between each pair of genes. Different genes with similar expression patterns across the different developmental stages fall into the same cluster, whereas dissimilar in expression genes fall in different clusters (D’haeseleer 2005; Altman and Krzywinski 2017). The complete linkage clustering method to cluster the genes hierarchically and the number of clusters was set to either four or six (*p_adj_*< 0.01). Cluster number was defined according to preliminary test runs (data not shown), which revealed that amongst the different clustering runs, 4-cluster and 6-cluster analyses generate sufficiently discernible expression patterns between cultivars, with enough high enrichment in oil production and fruit developmental genes. Within the 4- and 6-cluster analyses results, individual endocarp and fruit clusters were further investigated based on gene trend patterns between cultivars and high enrichment in the aforementioned genes (Figure 5 and Supplementary Data S8).Both gene expression trends and gene expression trends per cluster plots were created by the package tidyverse (<https://tidyverse.tidyverse.org/articles/paper.html>).

**GO enrichment analysis**

GO (Gene Ontology) enrichment analysis of the annotated gene lists generated in this study was performed using the g:GOSt tool of the g:Profiler web server (Raudvere et al. 2019). This online tool maps the given list of gene identifiers to functional information sources (e.g. Ensembl databases) and detects statistically significant term enrichment within the data. Moreover, in the analysis KEGG Reactome (<https://reactome.org/>) and WikiPathways (<https://www.wikipathways.org/>) are also included. The queries of the corresponding gene lists were run with the default parameters, using the Olea europaea var. sylvestris gene annotations, including KEGG pathway options. The generated GO term lists and the associated pathways were classified into three ontologies including MF (Molecular Functions), BP (Biological Processes), and CC (Cellular Components). For presentation purposes and to facilitate discussion, the retrieved GO enriched terms were summarized per query by removing redundant GO terms using the REVIGO online tool (http://revigo.irb.hr/).

**Identification of genes involved in oil production and fruit shape/ size development**

To identify genes involved in oil production and fruit development in the generated comparative transcriptomic data, the highly specific genes datasets (Supplementary Data S3), the flower, endocarp, and fruit CHO vs KO DEGs datasets (Supplementary Data S4), as well as the most significantly enriched CHO/KO co-expression gene clusters (Supplementary Data S6 and S8), were surveyed for the presence of “known” genes with documented function in these processes (Supplementary Data S9 and S10). The list of “known” genes was generated by retrieving entries from genome annotation databases, such as PLAZA 5.0 (<https://bioinformatics.psb.ugent.be/plaza/versions/plaza_v5_dicots/>), the Phytozome (<https://phytozome-next.jgi.doe.gov/>), and Cytochrome P450 (<https://drnelson.uthsc.edu/>), based on their documented description. Some entries in the list were also retrieved from related scientific publications.

**Quantitative real-time PCR (qRT-PCR) analysis**

To verify the RNA-seq results, five of the most significant and highly expressed CHO vs KO DEGs reported to be involved in oil production were analysed with qRT-PCR. cDNA was prepared from RNA pools of equimolarly mixed biological triplicates for each tissue type per cultivar using the SuperScript™ II Reverse Transcriptase kit (Thermo Fisher Scientific, Waltham, MA USA). Real-time PCR amplification was performed according to the SuperScript™ II Reverse Transcriptase (Thermo Fisher Scientific, Waltham, MA USA) instructions in a Rotor-Gene 6000 real-time 5-Plex HRM PCR Thermocycler (Corbett Research, Sydney, Australia), using the Rotor-Gene Q software version 2.0.2 (Corbett Life Science, Cambridge, UK). The reactions for each gene per cultivar and tissue type were performed in triplicates. The gene-specific primers were designed using Primer3 (http://bioinfo.ut. ee/primer3-0.4.0/) (Supplementary Table 6). The *O. europaea* L. polyubiquitin (*OUB2*) was used as an internal reference gene (Ray and Johnson 2014). Sample calibration was performed using either the CHO endocarp stage 2 or the CHO fruit stage 3 for normalization. Relative gene expression was calculated using the 2-ΔΔCt method (Livak and Schmittgen 2001). Statistical analysis of the qRT-PCR results was performed using the JASP (V.0.16.3) software (https://jasp-stats.org/).

**Database development**

To ensure that the availability of the complete dataset analysed is in accordance to the findability, accessibility, interoperability, and reusability (FAIR) principles, the raw sequences were published in the public database GenBank (BioProject ID: PRJNA763324; <https://www.ncbi.nlm.nih.gov/bioproject/?term=Olea>) and the analysed datasets were become available via the dedicated database GrOlivedb (https://grolivedb.com). The GrOlivedb is a web-based, curated, relational database developed using Tripal v3 (Spoor et al. 2019) that builds upon the open-source Drupal content management system and the GMOD Chado database schema (Mungall et al. 2007). In addition, Tripal extension modules, including, the Expression tool that allows the visual representation of the level of expression of specific genes in each tissue (https://github.com/tripal/tripal_analysis_expression.git), and the NCBI BLASTn tool (Altschul et al. 1997) that can execute sequence similarity searches within given nucleotide databases (Camacho et al. 2009), were implemented, enabling independent gene code (NCBI GenInfo Identifier) and nucleotide sequence-based gene searches and visualization. The expression tool utilized normalized gene count data for each tissue and developmental stage of both the "Chondrolia Chalkidikis" and the "Koroneiki" olive cultivars. On the other hand, the Blastn tool allows the sequence homology search within a nucleotide sequence database per cultivar, sequence which was derived by the SamTools (v. 1.16) integrated Bayesian "Gap5" consensus algorithm (Boulding et al. 1993), utilizing the transcriptomic heterozygous consensus sequence data of the mature leaf tissues of each cultivar and their mapping quality scores. The database web server is hosted on an inhouse Linux-dedicated server (Debian v10.12) with nginx v1.19.6, PostgreSQL v11.16, PHP v7.3.33, and Drush v8.1.18., Tripal 3, and Chado 1.3 (including Tripal Biomaterial).

**Supplementary results**

**GO enrichment analysis of CHO vs KO DEGs**

GO enrichment analysis of the fruit DEGs between the two cultivars revealed significant enrichment in protein phosphorylation (GO:0006468, *p_adj_*= 4.55E-16) and phosphorus metabolism (GO:0006793, *p_adj_*= 1.36E-12) at stage 2 fruits, as well as photosynthesis-related (GO:0015979, *p_adj_*= 4.02E-09) and phosphorylation-related (GO:0016310, *p_adj_*= 1.79E-10) biological processes for the mature fruit (stage 4) DEGs (Supplementary Table 3 and Supplementary Data S5). Accordingly, the endocarp stages showed highly significant enrichment in transmembrane transport (GO:0055085, *p_adj_*= 2.40E-09) and regulation of transcription (GO:0006355, *p_adj_*= 8.10E-08) for stage 2, carbohydrate metabolism and monocarboxylic acid biosynthesis for stage 3 (GO:0005975, *p_adj_*= 2.75E-11; GO:0072330, *p_adj_*= 5.98E-07) and stage 4 (GO:0005975, *p_adj_*= 8.46E-07; GO:0032787, *p_adj_*= 5.85337E-06), as well as protein phosphorylation (GO:0006468, *p_adj_*= 4.51E-07), and phosphorus metabolism (GO:0006793, *p_adj_*= 3.40E-07) for stage 4 endocarp DEGs (Supplementary Table 3 and Supplementary Data S5). GO enrichment analysis of the young leaf DEGs showed significant enrichment in biological processes, including translation (GO:0006412, *p_adj_*= 8.25E-31), peptide metabolism (GO:0006518, *p_adj_*= 2.66E-27), gene expression (GO:0010467, *p_adj_*= 4.45E-16), macromolecule biosynthesis (GO:0009059, *p_adj_*= 5.44E-10), and photosynthesis (GO:0015979, *p_adj_*= 1.18E-08) (Supplementary Table 3 and Supplementary Data S5). Pertaining to the young shoot DEGs, significant enrichment was observed for protein phosphorylation (GO:0006468, *p_adj_*= 3.08E-10), regulation of transcription (GO:0006355, *p_adj_*= 7.03935E-05), and movement of cell/ subcellular component (GO:0006928, *p_adj_*= 1.80E-07) (Supplementary Table 3 and Supplementary Data S5). On the contrary, the closed flower DEGs between the two cultivars showed the highest significant enrichment in microtubule and movement of cell/subcellular components biological processes (GO:0006928, *p_adj_*= 9.29E-12; GO:0007017, *p_adj_*= 8.64E-09) (Supplementary Table 3 and Supplementary Data S5). Finally, functional enrichment analysis of the root DEGs among the two cultivars showed significant enrichment in phosphorylation processes (GO:0006468, *p_adj_*= 1.36E-09), followed by regulation of transcription (GO:2001141, *p_adj_*= 3.62E-08), and protein modification processes (GO:0006355, *p_adj_*= 3.62E-08) (Supplementary Table 3 and Supplementary Data S5).

**GO enrichment analysis of the hierarchical clustering datasets (4-clusters)**

To reveal the biological processes present in the fruit and endocarp CHO/KO gene modules of the 4-cluster analysis, functional enrichment analysis was performed in gene clusters with differential expression patterns between cultivars. Concerning the fruit tissues, the GC2 and GC3 were selected for further analysis (Figure 5). The genes in GC2 showed significant enrichment in photosynthesis (GO:0015979, *p_adj_*= 4.83E-06) and beta-glucan metabolic processes (GO:0051273, *p_adj_*= 2.68E-02), while analysis of the GC3 revealed significant enrichment in photosynthesis-related processes (GO:0015979, *p_adj_*= 1.30E-02), protein repair (GO:0030091, *p_adj_*= 1.51E-02), and phosphorylation processes (GO:0006468, *p_adj_*= 7.96E-04) (Supplementary Table 4 and Supplementary Data S7). Overall, the GO enrichment analysis indicate that genes involved in protein repair, protein phosphorylation, cell-wall structure, and especially photosynthesis are differentially expressed with the onset of fruit development between the two cultivars.

Regarding the endocarp tissues, GC1, which contains genes that are up-regulated with endocarp maturation in CHO (Figure 5B), showed highly significant enrichment in protein synthesis related processes (GO:0006399, *p_adj_*= 9.36E-04; GO:0043038, *p_adj_*= 2.18E-06; GO:0006413, *p_adj_*= 2.41E-05) (Supplementary Table 4 and Supplementary Data S7). In contrast, the genes in GC3, which exhibit the opposite pattern (Figure 5B), showed significant enrichment in lipid metabolism (GO:0006629, *p_adj_*= 3.71E-03), positive regulation of transcription (GO:0045893, *p_adj_*= 5.53E-03), and microtubule-based processes (GO:0007017, *p_adj_*= 8.17E-03) (Supplementary Table 4 and Supplementary Data S7). Concerning GC4, although the functional enrichment analysis did not return any highly significant enrichment in BP GO terms, there was a significant enrichment in cellular components including photosystem II oxygen-evolving complex (GO:0009654, *p_adj_*= 5.59E-03) indicating an involvement of the genes in this cluster in photosynthesis-related processes (Supplementary Table 4 and Supplementary Data S7).

**GO enrichment analysis of the hierarchical clustering datasets (6-clusters)**

Functional enrichment analysis of the fruit GC2, which comprises of genes that show a maturation-dependent downregulation in CHO (Figure 5C), revealed their involvement in protein synthesis (GO:0006418, *p_adj_*= 3.32E-04), defense responses (GO:0006952, *p_adj_*= 5.26E-04), and phosphorus metabolism (GO:0006793, *p_adj_*= 8.03E-04) (Supplementary Table 5 and Supplementary Data S7). Conversely, the genes in the fruit GC4, which are upregulated in the more mature CHO fruit stages (Figure 5C), exhibited significant enrichment in amide metabolic processes (GO:0043603, *p_adj_*= 7.80E-27), as well as protein (GO:0042254, *p_adj_*= 1.80E-02) and nucleotide-related (GO:0009165, *p_adj_*= 4.57E-02) biosynthetic processes (Supplementary Table 5 and Supplementary Data S7). Enrichment analysis of the endocarp GC1 of the 6-cluster analysis revealed that the genes in this cluster are involved in several protein synthesis-related processes (GO:0006413, *p_adj_*= 9.85E-07; GO:0044272, *p_adj_*= 1.69E-02; GO:0043039, *p_adj_*= 2.40E-02) and monosaccharide metabolism (GO:0044281, *p_adj_*= 7.87E-14) (Supplementary Table 5 and Supplementary Data S7). Notably, the genes in GC3, which are downregulated in the mature CHO endocarp stages (Figure 5D), showed significant enrichment (GO:0006325, *p_adj_*= 5.95E-03) and RNA silencing mechanisms (GO:0031047, *p_adj_*= 1.63E-02) (Supplementary Table 5 and Supplementary Data S7). Finally, endocarp GC4 revealed enrichment only in protein phosphorylation-related processes (GO:0016310, *p_adj_*= 1.75E-02) (Supplementary Table 5 and Supplementary Data S7).

## Validation of DEGs data by qRT-PCR

The expression of several of the most significant and highly expressed CHO vs KO DEGs with reported role in oil production (Supplementary Data S9) were also analysed by qRT-PCR to verify the validity of the RNA-seq results. The analysis involved the 3-oxoacyl-[acyl-carrier protein] reductase (Oeu027948.1), enoyl reductase (Oeu015278.1), and acyl-CoA oxidase (Oeu015636.1) genes that showed high fold differential gene expression in the CHO tissues, as well as the malate dehydrogenase genes (Oeu005735.1, Oeu061514.1) with high fold change in the KO tissues (Supplementary Figure 3). The expression profile of the tested DEGs between cultivars was in accordance with the RNA-seq data (Supplementary Figure 3 and Supplementary Data S9). More specifically, the CHO highly expressed genes (Oeu027948.1 Oeu015278.1, and Oeu015636.1) showed higher relative expression values in the tested CHO tissues, while the malate dehydrogenase genes (Oeu005735.1, and Oeu061514.1) showed higher fold change in the KO tissues (Supplementary Figure 3). Notably, the differential expression of the malate dehydrogenase gene (Oeu061514.1), which showed higher fold change in most of the KO tissues except the closed flower (Supplementary Data S9), was also reflected in the qRT-PCR analysis, exhibiting higher fold change value in the CHO closed flower tissue in contrast to KO (Supplementary Figure 3).

**Supplementary Tables and Figures**

**Supplementary Table 1.** Tissue samples of the olive tree cultivars “Chondrolia Chalkidikis” (CHO) and “Koroneiki” (KO) used in this work. All samples were collected in three biological replicates per tissue type and developmental stage.

| **No.** | **Organ/ Tissue type** | **Developmental stage*** |
| --- | --- | --- |
| 1 | Mature leaves | 19 |
| 2 | Young leaves | 11 |
| 3 | Mature shoots | 19 |
| 4 | Young shoots | 11 |
| 5 | Closed flower | 57 |
| 6 | Open flower | 65 |
| 7 | 2^nd^ stage Fruit (epicarp plus mesocarp) | 79 |
| 8 | 3^rd^ stage Fruit (epicarp plus mesocarp) | 81 |
| 9 | 4^th^ stage Fruit (epicarp plus mesocarp) | 89 |
| 10 | 4^th^ stage Stalk | 89 |
| 11 | 2^nd^ stage Endocarp | 79 |
| 12 | 3^rd^ stage Endocarp | 81 |
| 13 | 4^th^ stage Endocarp | 89 |
| 14 | Root | - |

* as described by (Sanz-Cortés et al. 2002)

**Supplementary Table 2.** Comparison of mapping results between olive genome references. The mapping accuracy differs between cultivars.

| ***O. europaea* var. *sylvestris* (wild olive)** | | | |
| --- | --- | --- | --- |
| **CHO_mature_leaf** | | | |
| Number of input reads | 12643156 | 12995232 | 17067991 |
| Uniquely mapped reads number | 10511470 | 10968981 | 14397238 |
| Percentage of uniquely mapped reads | 83.13961 | 84.40774 | 84.35227 |
| **KO_mature_leaf** | | | |
| Number of input reads | 28631486 | 30428271 | 34011558 |
| Uniquely mapped reads number | 23563769 | 24961968 | 27995908 |
| Percentage of uniquely mapped reads | 82.3002 | 82.03545 | 82.31292 |
|  | | | |
| ***O. europaea* subsp. *europaea* (common olive-OLEA9)** | | | |
| **CHO_mature_leaf** | | | |
| Number of input reads | 12643156 | 12995232 | 17067991 |
| Uniquely mapped reads number | 10253010 | 11307970 | 14268095 |
| Percentage of uniquely mapped reads | 81.09534 | 87.0163 | 83.59563 |
| **KO_mature_leaf** | | | |
| Number of input reads | 28631486 | 30428271 | 34011558 |
| Uniquely mapped reads number | 24711443 | 26242735 | 29401481 |
| Percentage of uniquely mapped reads | 86.30863 | 86.24458 | 86.44556 |

**Supplementary Table 3.** The most highly significant GO terms enriched in the CHO vs KO DEGs datasets for indicative tissue types/ developmental stages. The complete list of the identified GO terms per tissue type can be found in Supplementary Data S5.

| **Tissue type** | **GO term** | **Biological process** |
| --- | --- | --- |
| Fruit (2nd stage) | GO:0006468 | Protein phosphorylation |
|  | GO:0006793 | Phosphorus metabolism |
| Fruit (4th stage) | GO:0015979 | Photosynthesis-related |
|  | GO:0016310 | Phosphorylation-related |
| Endocarp (2nd stage) | GO:0055085 | Transmembrane transport |
|  | GO:0006355 | Regulation of transcription |
| Endocarp (3rd stage) | GO:0005975 | Carbohydrate metabolism |
|  | GO:0072330 | Monocarboxylic acid biosynthesis |
| Endocarp (4th stage) | GO:0032787 | Monocarboxylic acid metabolism |
|  | GO:0005975 | Carbohydrate metabolism |
|  | GO:0006468 | Protein phosphorylation |
|  | GO:0006793 | Phosphorus metabolism |
| Young leaf | GO:0006412 | Translation |
|  | GO:0006518 | Peptide metabolism |
|  | GO:0010467 | Gene expression |
|  | GO:0009059 | Macromolecule biosynthesis |
|  | GO:0015979 | Photosynthesis |
| Young shoot | GO:0006468 | Protein phosphorylation |
|  | GO:0006355 | Regulation of transcription |
|  | GO:0006928 | Movement of cell/ subcellular component |
| Closed flower | GO:0007017 | Microtubule |
|  | GO:0006928 | Movement of cell/ subcellular components |
| Root | GO:0006468 | Phosphorylation processes |
|  | GO:2001141 | Regulation of transcription |
|  | GO:0036211 | Protein modification processes |

**Supplementary Table 4.** The most significantly enriched GO terms in gene clusters with differential expression patterns between cultivars (4-cluster analysis). The complete list of the identified GO terms per gene cluster can be found in Supplementary Data S7.

| **Tissue type** | **Gene cluster** | **GO term** | **Biological process/ Cellular component** |
| --- | --- | --- | --- |
| Fruit | GC2 | GO:0015979 | Photosynthesis |
|  |  | GO:0051273 | Beta-glucan metabolic processes |
|  | GC3 | GO:0015979 | Photosynthesis-related processes |
|  |  | GO:0030091 | Protein repair |
|  |  | GO:0006468 | Phosphorylation |
| Endocarp | GC1 | GO:0006399 | tRNA metabolic processes |
|  |  | GO:0043038 | Amino acid activation |
|  |  | GO:0006413 | Translational initiation |
|  | GC3 | GO:0006629 | Lipid metabolism |
|  |  | GO:0007017 | Microtubule-based processes |
|  |  | GO:0045893 | Positive regulation of transcription |
|  | GC4 | GO:0009654 | Photosystem II oxygen-evolving complex |

**Supplementary Table 5.** The most significantly enriched GO terms in gene clusters with differential expression patterns between cultivars (6-cluster analysis). The complete list of the identified GO terms per gene cluster can be found in Supplementary Data S7.

| **Tissue type** | **Gene cluster** | **GO term** | **Biological process** |
| --- | --- | --- | --- |
| Fruit | GC2 | GO:0006418 | tRNA aminoacylation for protein translation |
|  |  | GO:0019752 | carboxylic acid metabolic process |
|  |  | GO:0006952 | defence response |
|  |  | GO:0006793 | phosphorus metabolic process |
|  | GC4 | GO:0043603 | amide metabolic process |
|  |  | GO:0051641 | cellular localization |
|  |  | GO:0042254 | ribosome biogenesis |
|  |  | GO:0009165 | nucleotide biosynthetic process |
| Endocarp | GC1 | GO:0044281 | small molecule metabolic process |
|  |  | GO:0006413 | translational initiation |
|  |  | GO:0044272 | sulphur compound biosynthetic process |
|  |  | GO:0043039 | tRNA aminoacylation |
|  | GC3 | GO:1901617 | organic hydroxy compound biosynthetic process |
|  |  | GO:0006325 | chromatin organization |
|  |  | GO:0009081 | branched-chain amino acid metabolic process |
|  |  | GO:0031047 | RNA-mediated gene silencing |
|  | GC4 | GO:0016310 | phosphorylation |
|  |  | GO:0006468 | protein phosphorylation |

**Supplementary Table 6.** Gene-specific qRT-PCR primers used in this study.

| **Name** | **Sequence (5' to 3'):** | **Length** | **%GC** | **Tm** | **TM calculator** |
| --- | --- | --- | --- | --- | --- |
| Oeu005735.1_32 F | TGAAGCGAGCTGAATGCAGA | 20 | 50 | 60 | 55 |
| Oeu005735.1_231 R | CACCACAGCACCAGTATCCA | 20 | 55 | 59.7 |  |
| Oeu015278.1_113 F | TGAAAGGGAAGCACATGCAG | 20 | 50 | 58.8 | 54 |
| Oeu015278.1_312 R | CGTGATGGCTGAACGTAGCA | 20 | 55 | 60.7 |  |
| Oeu015636.1_1,297 F | GAATTGTGGGGCGAAGATGC | 20 | 55 | 59.9 | 54 |
| Oeu015636.1_1,496 R | GTTGGTGCAAGCTCAAAGGA | 20 | 50 | 59 |  |
| Oeu027948.1_186 F | TTGGAGCCATGGCAGGAATT | 20 | 50 | 60 | 54 |
| Oeu027948.1_385 R | TGGCTCGCATCATTCGATCT | 20 | 50 | 59.6 |  |
| Oeu061514.1_186 F | CCATGATTGCTAGAGGGGCC | 20 | 60 | 60.3 | 55 |
| Oeu061514.1_385 R | ACCAACCATGACGGCAATGT | 20 | 50 | 60.5 |  |


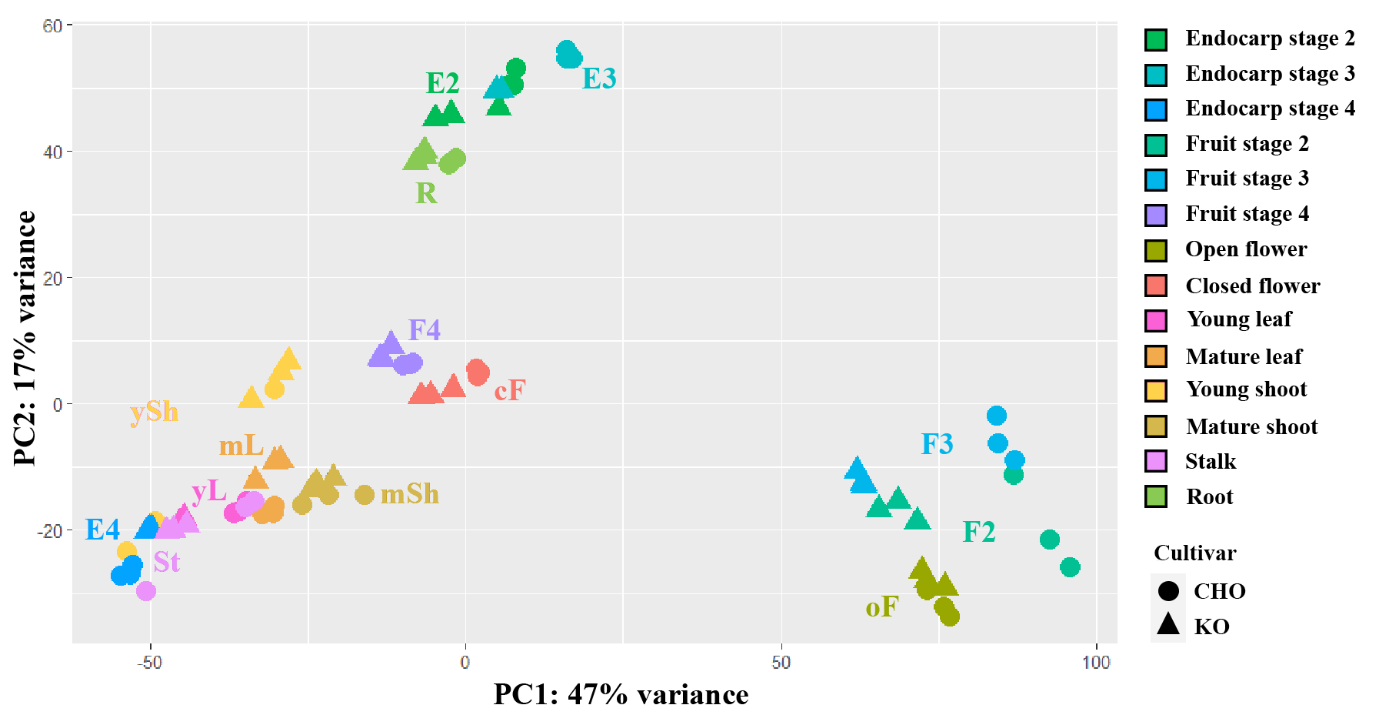
**Supplementary Figure 1.** Principal Component Analysis (PCA) of gene expression amongst 14 tissue types/ developmental stages for both CHO and KO cultivars. The triplicates are shown independently for each tissue type/ developmental stage per cultivar. Samples were annotated based on colour as an indication of tissue type and shape for indicating cultivar. E2: endocarp stage 2, E3: endocarp stage 3, E4: endocarp stage 4, F2: fruit stage 2, F3: fruit stage 3, F4: fruit stage 4, oF: open flower, cF: closed flower, yL: young leaf, mL: mature leaf, ySh: young shoot, mSh: mature shoot, St: stalk, and R: root.


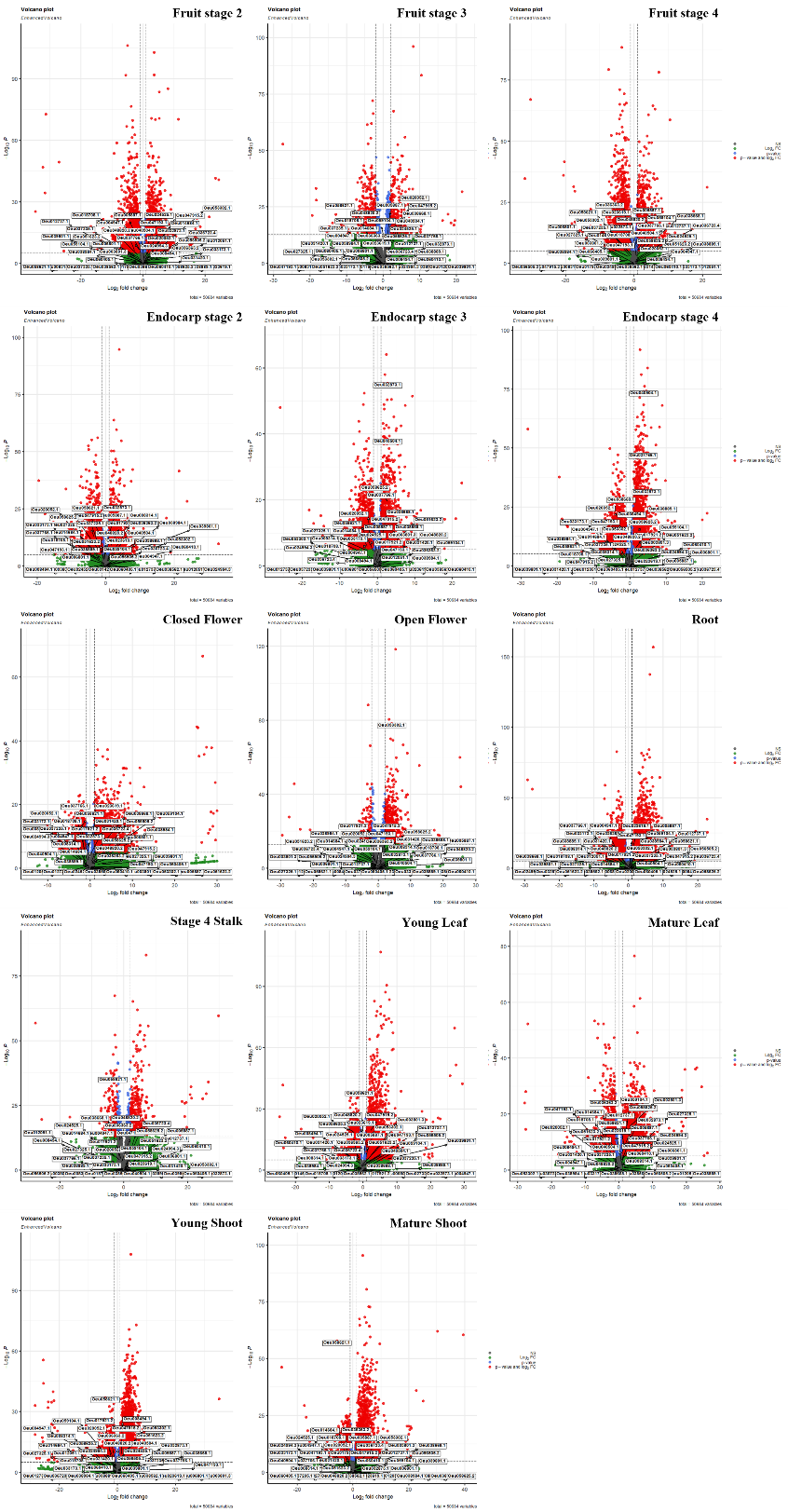
**Supplementary Figure 2.** Differential gene expression of CHO vs KO tissues/ developmental stages. The volcano plots depict DEGs of stage 2-4 fruit, stage 2-4 endocarp, closed and open flower, young and mature leaf, young and mature shoot, root, as well as stalk tissues of the CHO in contrast to the KO cultivar. Color-coding was used to signify DEGs with high statistical significance (*p_adj_*≤ 0.05) and high Log_2_ Fold Change (1> FC <-1) (red), high statistical significance but low fold change (blue), and no significant DEGs with either high or low fold change (green and blue, respectively). Annotations correspond to genes with biological interest.


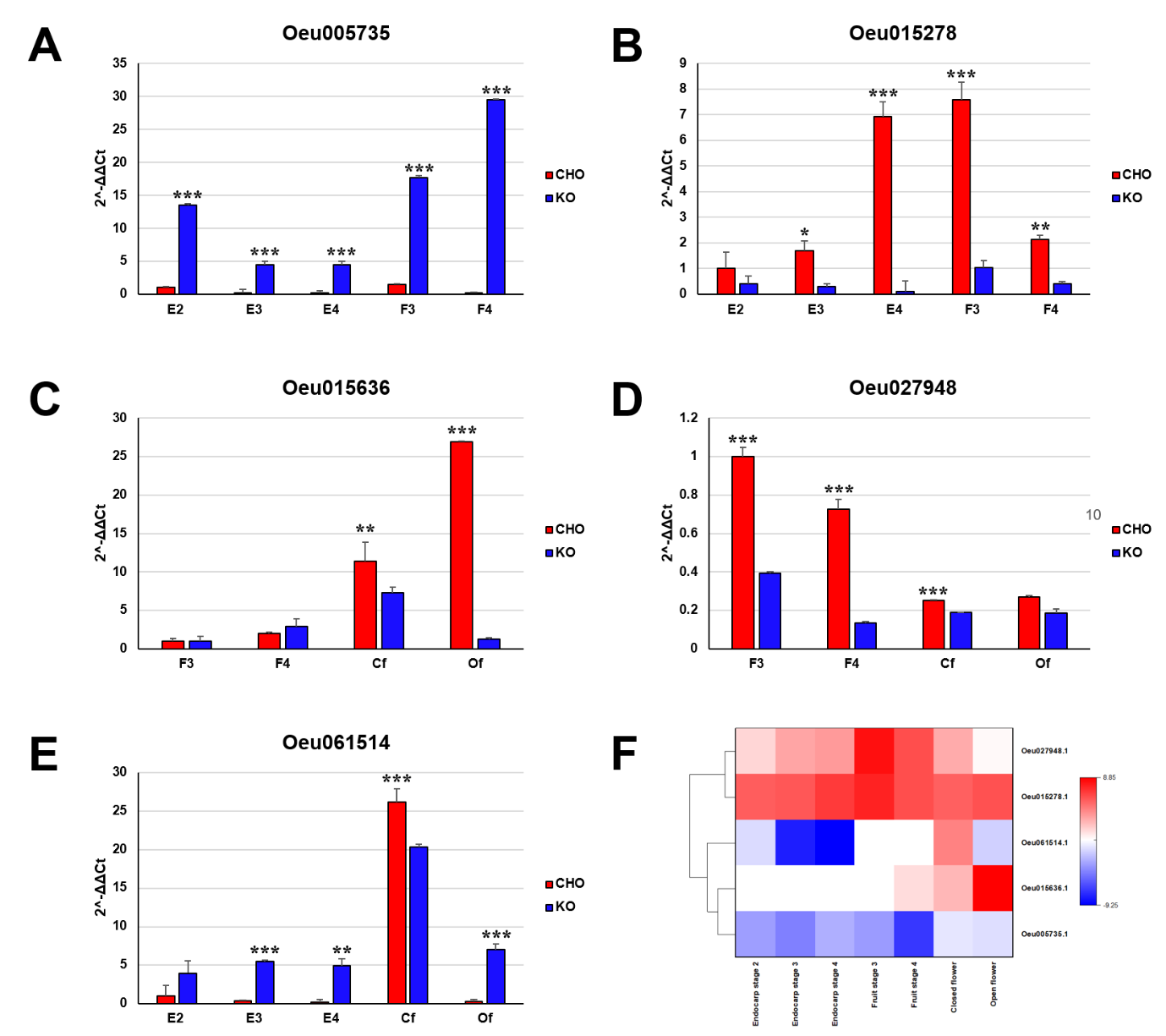
**Supplementary Figure 3.** qRT-PCR validation of five significant highly expressed CHO vs KO DEGs known to be involved in fatty acid metabolism. (A-E) Barplots depicting the average relative expression (2^-ΔΔCt^) of malate dehydrogenase [Oeu005735.1, Oeu061514.1] (A, E), enoyl reductase [Oeu015278.1] (B), acyl-CoA oxidase [Oeu015636.1] (C), and 3-oxoacyl-[acyl-carrier protein] reductase [Oeu027948.1] (D) genes in CHO and KO tissues. The CHO Endocarp stage 2 (A, B, and E) and Fruit stage 3 (C and D) were used as the calibrator samples. All samples were analysed in triplicates. Error bars represent standard deviation of three technical replicates. Asterisks indicate the significance of relative gene expression between cultivars for each respective tissue, with ^*^ *p_tukey_*≤ 0.05, ^**^ *p_tukey_*< 0.01, and ^***^ *p_tukey_*< 0.001. E2= Endocarp stage 2, E3= Endocarp stage 3, E4= Endocarp stage 4, F3= Fruit stage 3, F4= Fruit stage 4, Cf= Closed flower, and Of= Open flower. (F) Hierarchical clustering heatmap demonstrating the CHO vs KO differential gene expression of the analysed genes for endocarp, fruit, and flower tissues. Data was derived from Supplementary Data S9. The color-scale legend represents Log_2_ Fold Change (FC) values, with red (Log_2_ FC> 0) indicating higher CHO and blue (Log_2_ FC< 0) higher KO expression.

**References**

Altman N, Krzywinski M (2017) Points of Significance: Clustering. Nature Methods 14: 545–546

Altschul SF, Madden TL, Schäffer AA, Zhang J, Zhang Z, Miller W, Lipman DJ (1997) Gapped BLAST and PSI-BLAST: a new generation of protein database search programs. Nucleic Acids Res 25: 3389–3402

Anders S, Huber W (2010) Differential expression analysis for sequence count data. Genome Biology 11:

Bazakos C, Alexiou KG, Ramos-Onsins S, Koubouris G, Tourvas N, Xanthopoulou A, Mellidou I, Moysiadis T, Vourlaki I-T, Metzidakis I, Sergentani C, Manolikaki I, Michailidis M, Pistikoudi A, Polidoros A, Kostelenos G, Aravanopoulos F, Molassiotis A, Ganopoulos I (2023) Whole genome scanning of a Mediterranean basin hotspot collection provides new insights into olive tree biodiversity and biology. The Plant Journal 116: 303–319

Boulding W, Kalra A, Staelin R, Zeithaml VA (1993) A Dynamic Process Model of Service Quality: From Expectations to Behavioral Intentions. Journal of Marketing Research 30: 7–27

Camacho C, Coulouris G, Avagyan V, Ma N, Papadopoulos J, Bealer K, Madden TL (2009) BLAST+: architecture and applications. BMC bioinformatics 10:

D’haeseleer P (2005) How does gene expression clustering work? NATURE BIOTECHNOLOGY 23:

Dobin A, Davis CA, Schlesinger F, Drenkow J, Zaleski C, Jha S, Batut P, Chaisson M, Gingeras TR (2013) STAR: ultrafast universal RNA-seq aligner. Bioinformatics 29: 15–21

Jiménez-Ruiz J, Ramírez-Tejero JA, Fernández-Pozo N, Leyva-Pérez M de la O, Yan H, Rosa R de la, Belaj A, Montes E, Rodríguez-Ariza MO, Navarro F, Barroso JB, Beuzón CR, Valpuesta V, Bombarely A, Luque F (2020) Transposon activation is a major driver in the genome evolution of cultivated olive trees (Olea europaea L.). The Plant Genome 13: e20010–e20010

Julca I, Marcet-Houben M, Cruz F, Gómez-Garrido J, Gaut BS, Díez CM, Gut IG, Alioto TS, Vargas P, Gabaldón T (2020) Genomic evidence for recurrent genetic admixture during the domestication of Mediterranean olive trees (Olea europaea L.). BMC Biology 2020 18:1 18: 1–25

Livak KJ, Schmittgen TD (2001) Analysis of Relative Gene Expression Data Using Real-Time Quantitative PCR and the 2−ΔΔCT Method. Methods 25: 402–408

Love MI, Huber W, Anders S (2014) Moderated estimation of fold change and dispersion for RNA-seq data with DESeq2. Genome biology 15:

Mungall CJ, Emmert DB, Gelbart WM, de Grey A, Letovsky S, Lewis SE, Rubin GM, Shu SQ, Wiel C, Zhang P, Zhou P (2007) A Chado case study: an ontology-based modular schema for representing genome-associated biological information. Bioinformatics (Oxford, England) 23:

Rao G, Zhang J, Liu X, Lin C, Xin H, Xue L, Wang C (2021) De novo assembly of a new Olea europaea genome accession using nanopore sequencing. Horticulture Research 8:

Raudvere U, Kolberg L, Kuzmin I, Arak T, Adler P, Peterson H, Vilo J (2019) G:Profiler: A web server for functional enrichment analysis and conversions of gene lists (2019 update). Nucleic Acids Research 47: W191–W198

Ray DL, Johnson JC (2014) Validation of reference genes for gene expression analysis in olive (Olea europaea) mesocarp tissue by quantitative real-time RT-PCR. BMC research notes 7:

Reynolds AP, Richards G, De La Iglesia B, Rayward-Smith VJ (2006) Clustering Rules: A Comparison of Partitioning and Hierarchical Clustering Algorithms. Journal of Mathematical Modelling and Algorithms 2006 5:4 5: 475–504

Sanz-Cortés F, Martinez-Calvo J, Badenes ML, Bleiholder H, Hack H, Llacer G, Meier U (2002) Phenological growth stages of olive trees (Olea europaea). Annals of Applied Biology 140: 151–157

Spoor S, Cheng CH, Sanderson LA, Condon B, Almsaeed A, Chen M, Bretaudeau A, Rasche H, Jung S, Main D, Bett K, Staton M, Wegrzyn JL, Feltus FA, Ficklin SP (2019) Tripal v3: an ontology-based toolkit for construction of FAIR biological community databases. Database: The Journal of Biological Databases and Curation 2019: 77

Tourvas N, Ganopoulos I, Koubouris G, Kostelenos G, Manthos I, Bazakos C, Stournaras V, Molassiotis A, Aravanopoulos F (2023) Wild and cultivated olive tree genetic diversity in Greece: a diverse resource in danger of erosion. Front Genet 14:

Unver T, Wu Z, Sterck L, Turktas M, Lohaus R, Li Z, Yang M, He L, Deng T, Escalante FJ, Llorens C, Roig FJ, Parmaksiz I, Dundar E, Xie F, Zhang B, Ipek A, Uranbey S, Erayman M, Ilhan E, Badad O, Ghazal H, Lightfoot DA, Kasarla P, Colantonio V, Tombuloglu H, Hernandez P, Mete N, Cetin O, Van Montagu M, Yang H, Gao Q, Dorado G, Van de Peer Y (2017) Genome of wild olive and the evolution of oil biosynthesis. Proceedings of the National Academy of Sciences of the United States of America 114: E9413–E9422

Yanai I, Benjamin H, Shmoish M, Chalifa-Caspi V, Shklar M, Ophir R, Bar-Even A, Horn-Saban S, Safran M, Domany E, Lancet D, Shmueli O (2005) Genome-wide midrange transcription profiles reveal expression level relationships in human tissue specification. Bioinformatics 21: 650–659
